# The sham effect of invasive interventions in chronic coronary syndromes: a systematic review and meta-analysis

**DOI:** 10.1186/s12872-022-02658-x

**Published:** 2022-05-14

**Authors:** Catarina Palma, Cláudio David, Ricardo M. Fernandes, Fausto J. Pinto, João Costa, Joaquim J. Ferreira, Daniel Caldeira

**Affiliations:** 1 Faculdade de Medicina, Universidade de Lisboa, Lisbon, Portugal; 2 Laboratory of Clinical Pharmacology and Therapeutics, Faculdade de Medicina, Universidade de Lisboa, Lisbon, Portugal; 3 Instituto de Medicina Molecular, Faculty of Medicine, Faculdade de Medicina, Universidade de Lisboa, Lisbon, Portugal; 4 Centro Cardiovascular da Universidade de Lisboa (CCUL@RISE), Faculdade de Medicina, Universidade de Lisboa, Lisbon, Portugal; 5 Department of Pediatrics, Santa Maria Hospital, Centro Hospitalar Univesitário Lisboa Norte (CHULN), Centro Académico de Medicina de Lisboa (CAML), Lisbon, Portugal; 6 Cardiology Department, Hospital Santa Maria, Centro Hospitalar Univesitário Lisboa Norte (CHULN), Centro Académico de Medicina de Lisboa (CAML), Lisbon, Portugal; 7 CNS – Campus Neurológico Sénior, Torres Vedras, Portugal

**Keywords:** Chronic coronary syndromes, Sham effect, Invasive treatment, Sham procedure

## Abstract

**Background:**

Some patients with chronic coronary syndromes undergo invasive procedures but the efficacy of such interventions remains to be robustly established by randomised sham-controlled trials (RCTs).

**Purpose:**

To determine the sham effect in patients with chronic coronary syndromes enrolled in RCTs by performing a systematic review and meta-analysis.

**Methods:**

In April 2022, we performed a literature search for published patient-blind RCTs (CENTRAL, MEDLINE®, PsycINFO, and reference lists) with sham procedures, reporting the pre-post effects in the invasive sham arm among patients with Canadian cardiovascular society (CCS) angina or angina equivalents.

**Results:**

16 RCTs were included with 546 patients in the sham arm. Pooled results showed that sham interventions were associated with: improvement of 7% (95% CI 2–11%; I^2^ = 0%) in exercise time; decrease of 0.78 (95% CI − 1.10 to − 0.47; I^2^ = 75%) in CCS angina class; decrease of 53% (95% CI 24–71%; I^2^ = 96%) and 25% (95% CI 20–29%; I^2^ = 0%) in anginal episodes and nitroglycerine (NTG) use, respectively. Pooled results also showed an improvement in the physical functioning, angina frequency, treatment satisfaction, and disease perception domains of the Seattle Angina Questionnaire (SAQ).

**Conclusion:**

Sham interventions in patients with chronic coronary syndromes were associated with a significant decrease in anginal episodes, NTG use, and CCS angina class and increased SAQ quality of life and exercise time. These results highlight the need for previous non sham-controlled trials to be interpreted with caution, and the importance of new invasive interventions to be evaluated versus a sham procedure.

**Supplementary Information:**

The online version contains supplementary material available at 10.1186/s12872-022-02658-x.

## Background

There are diverse clinical scenarios of chronic coronary syndromes that comprise both symptomatic and asymptomatic patients who have been diagnosed or suspected to have coronary artery disease (CAD). Symptomatic patients may present with angina (due to CAD, vasospastic, or microvascular disease) and/or other angina equivalent symptoms, as well as heart failure related with CAD [1]. A recent review of studies published between 2010 and 2017 estimated the worldwide prevalence of CAD to be approximately 5–8% [2].

The mainstays of treatment for chronic coronary syndromes are antiplatelet agents and aggressive management of risk factors to improve prognosis, and antianginal drugs for symptom relief. Certain patients undergo invasive treatments such as revascularisation procedures (either percutaneous coronary intervention [PCI] or coronary artery bypass graft [CAGB]) depending on the extent of ischaemia, severity, and location of the coronary plaques and/or due to how refractory symptoms are to medical treatment [1, 3, 4]. However, these interventions have not been shown to offer prognostic efficacy compared with sham interventions [5]. Throughout the history of CAD, remarkable benefits of certain interventions have been reported in non-controlled clinical trials, later failing to be shown efficacious against placebo in controlled trials. This emphasises the importance of placebo-controlled studies in recommending efficacious interventions [6].

In 1961, Shapiro defined the term placebo as any therapeutic procedure which, either deliberately or unknowingly, has an effect on a patient, symptom, syndrome, or disease but which is objectively without specific activity for the condition being treated [7]. Nowadays, the term placebo is commonly used to describe inactive pills. For other types of interventions, when the actual procedure is mimicked, it is called a sham procedure.

Angina is the most representative symptom of CAD and, along with exercise time, is often evaluated in clinical trials of chronic coronary syndromes. However, these types of outcomes are prone to the Hawthorne effect whereby a participant modifies their behaviour in reaction to being enrolled in a study and interacting with clinical trial staff, resulting in a possible enhancement of the placebo/sham effect.

In this systematic review, we aimed to evaluate and quantify the magnitude of the sham effect associated with invasive procedures among patients with chronic coronary syndromes.

## Methods

This systematic review and meta-analysis followed the Preferred Reporting Items for Systematic Reviews and Meta-Analyses guidelines for reporting systematic reviews evaluating healthcare interventions [8]. The protocol was registered with PROSPERO (reference: CRD42021224700).

### Eligibility criteria

For inclusion, we considered published randomised, patient-blind, controlled clinical trials (RCTs) with a sham procedure. A sham procedure is defined here as a procedure performed as a control that is similar to but omits a key therapeutic element of the treatment under investigation. RCTs were required to evaluate invasive treatments (PCI, CABG, coronary sinus reducer, myocardial laser revascularisation, or intramyocardial/coronary injection) for chronic coronary syndromes in patients with Canadian Cardiovascular Society (CCS) class I-IV angina, including refractory angina and angina equivalents. We excluded observational studies, conference abstracts, and congresses proceedings.

Our primary outcome was the relative change (measured as a percentage) in exercise time as recorded in a standardised exercise stress test. Secondary outcomes were the standardised mean differences in exercise time, CCS angina class, number of anginal episodes per week, nitroglycerine use (NTG) per week, and quality of life (QoL) assessment using the Seattle Angina Questionnaire (SAQ) [9]. The SAQ is a sensitive and specific instrument that includes a measure of QoL in CAD, the score ranges between 0 and 100, with higher scores indicating better function. To be included, studies had to report the pre-post difference in the sham procedure arm for at least one of the outcomes of interest.

### Information sources and search method

An electronic search for potentially eligible studies was performed in April 2022 using the Cochrane Central Register of Controlled Trials, MEDLINE®, and PsycINFO. OpenGrey was also searched. The search strategy is outlined in the Additional file 1. No language restrictions were applied. The reference lists of included studies and other literature reviews were also examined.

### Study selection, data collection process, and risk of bias assessment

Two authors independently screened titles and abstracts of the retrieved records. To ensure that the inclusion criteria were met, the full-text versions of the selected studies were assessed. Two authors independently extracted study characteristics and outcomes into a pre-established data collection form. Any disagreements were resolved through consensus. The reasons for exclusion were recorded at the full-text screening stage (see supplementary material).

Whenever data were only available in a plot, these were retrieved using the Plot Digitizer V.2.6.8. When studies presented different estimates for the outcome of interest, we extracted the most precise or adjusted measures. If an outcome was reported as a median value and interquartile range, we converted these values to mean ± standard deviations (SD) using the Wan Method [10]. If an outcome was reported as a mean change ± SD, we converted this to mean, lower, and upper confidence intervals. Mean ± standard errors were converted to mean ± SD. If data were obtained over several follow-ups, we analysed the estimate from putative sham/placebo effects as the most favourable effect registered in the follow-up.

The risk of bias was independently evaluated by two authors using the Cochrane risk of bias tool [11], whereby answering signalling questions in five domains enables an algorithm to generate a risk of bias judgement such as “Low risk”, “High risk” or “Some Concerns”.

All disagreements between reviewers throughout the different steps of the systematic review were resolved by consensus or by the decision of a third independent reviewer.

### Statistical analysis

Software Review Manager (RevMan) Version 5.4.1, Copenhagen: The Nordic Cochrane Centre, The Cochrane Collaboration, 2020 was used to obtain the estimates of individual studies, the pooled analysis, and to retrieve the forest plots.

Our first approach for the outcomes exercise time, anginal episodes, and nitroglycerine administration was to convert the absolute post-sham values into relative change (RC) values (RC was obtained by dividing the absolute value reported after the sham intervention by the absolute value reported before the sham intervention) compared with the baseline. Relative measures appear to be more stable than absolute measures across populations of patients who have different occurrence rates of the outcome under evaluation.

Next, we calculated the mean difference (MD) of the outcomes (MD was obtained by subtracting the absolute value after the sham intervention with the absolute value reported before the intervention) and 95% confidence intervals (95% CI) to estimate pooled results. While several studies assessed exercise time, this outcome was measured in a variety of ways, we, therefore, estimated the results for this outcome according to the measurement method and calculated the standardised mean difference (SMD) and 95% CI. Cohen's rule of thumb for effect size was used, with 0.2 considered a small effect, 0.5 a moderate effect, and 0.8 a large effect. Meta-analyses used random-effects models to pool the results.

Heterogeneity was assessed with the I^2^ test, which measures the percentage of total variation attributed to inter-study heterogeneity rather than chance [12]. The inverse of variance method with random-effects model was used by default, independently of the existence or not of statistical heterogeneity between study results, as we anticipated the inclusion of studies with different clinical and methodological characteristics.

We also planned to conduct subgroup analyses according to the type of sham procedure, mean left ventricular ejection fraction (< 50% or more than 33% of patients with heart failure vs. ≥ 50%/LVEF not reported), as well as a sensitivity analysis by excluding studies at a higher risk of bias. When feasible, meta-regression was performed for primary outcomes against age, the proportion of men, and the proportion of diabetic patients. Bubble graphs were plotted with STATA 17.0. The Jackknife leave-one-out sensitivity analysis [13] was also performed to evaluate the impact of a single study as responsible for the heterogeneity.

Publication bias was assessed through visual inspection of funnel plot asymmetry with Egger’s test [14]. If a small-study effect was suspected by visual inspection of the funnel plot or Egger’s test results, we planned to follow the trim and fill method to assess publication/small-study effects bias in the meta-analysis [15].

We used the Grading of Recommendations, Assessment and Evaluation (GRADE) framework to report the overall quality of evidence for primary outcomes [16]. The GRADE approach was independently assessed by two investigators and discrepancies were solved by consensus.

## Results

### Included studies

**Fig. 1:**
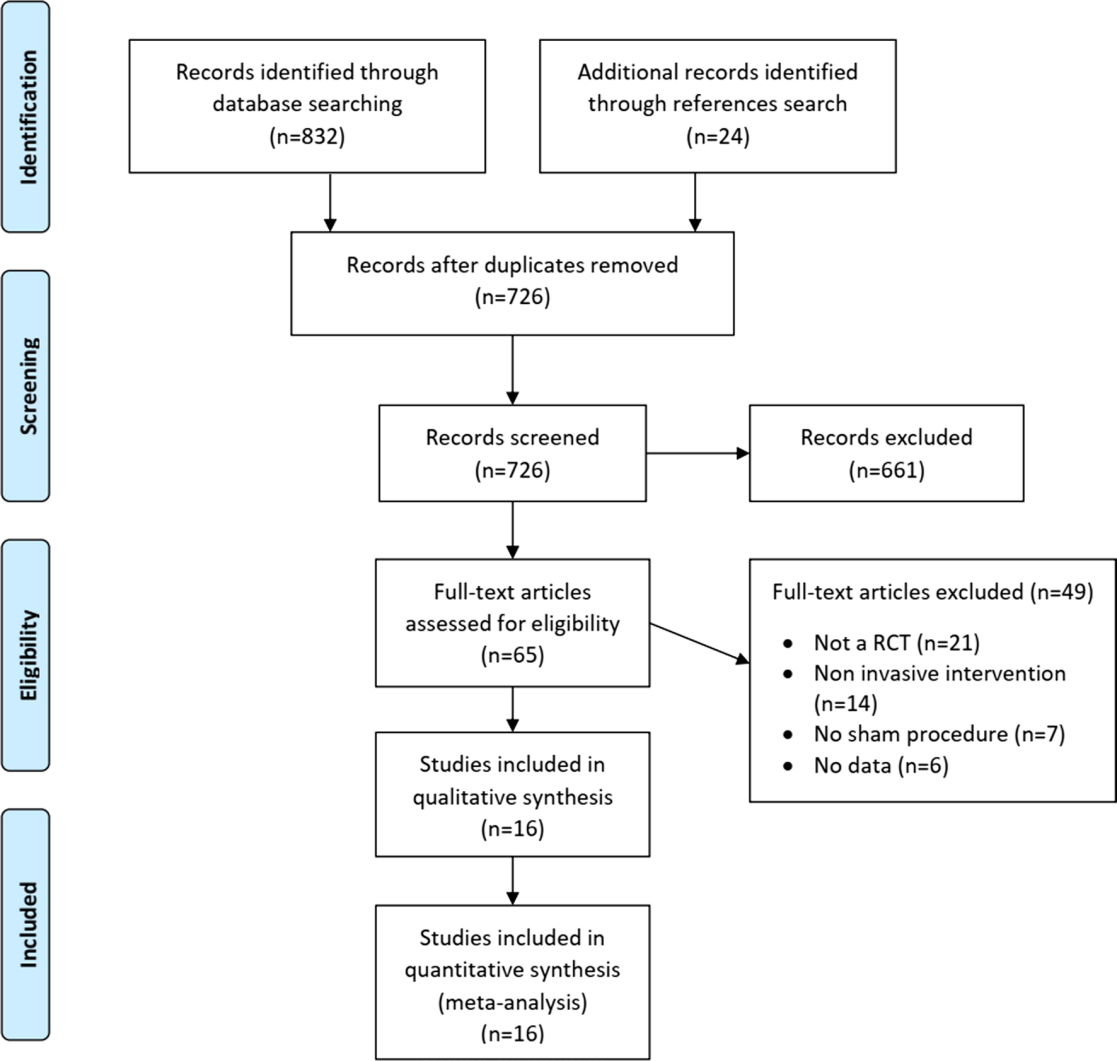
Study flow diagram

A total of 16 articles were retained for both qualitative and quantitative syntheses. No unpublished studies were retrieved (Fig. 1).

Sixteen randomised controlled trials were included with a total of 1340 participants (sample sizes between 10 and 298), in which 546 were allocated to the sham arm [5, 17–31]. Table 1 shows the characteristics of the included studies. Publication dates ranged from 1959 to 2017. All studies included patients with angina or equivalent symptoms. The mean age of the patients ranged from 57.8 to 67.8 years and the percentage of men ranged from 51 to 91%.

**Table 1:** Characteristics of the included randomised controlled trials (n = 16)

Name of study (author + year) Trial	Design	N (total)	Patients	Mean age Male (%)	Intervention	Comparator	Exercise protocol	Primary outcome	Included outcomes	Patients with HF or rEF (%)	Mean LVEF (%)
Al-Lamee 2017 ORBITA [5]	RCT (double-blind)	200	Patients with angina or equivalent symptoms and at least one angiographically significant lesion (≥ 70%) in a single vessel that was clinically appropriate for PCI	66 73%	PCI	Sham	Bruce protocol	Exercise time	Exercise time, SAQ	4	NR
Cobb [17]	RCT (double-blind)	17	Patients with seriously limited angina attributed to coronary artery disease	59 71%	Internal mammary artery ligation	Sham	Bruce protocol	NTG use and exercise time	Exercise time	NR	NR
Fuchs [22]	RCT (double-blind)	10	Patients with severe or critical stable angina pectoris (CCS class III or IV) despite maximal medical therapy and had no coronary artery revascularisation alternatives	65 90%	Percutaneous intramyocardial delivery of AdVEGF121	Diluent (placebo)	Treadmill using the ACIP protocol	Exercise time	Exercise time, CCS angina class, SAQ – angina frequency	NR	53.6
Kastrup 2005 Euroinject One [21]	RCT (double-blind)	80	Patients with severe stable ischaemic heart disease and CCS class III to IV	61 84%	Percutaneous intramyocardial plasmid gene transfer of phVEGF-A165	Placebo plasmid	NR	Myocardial perfusion defects during stress and rest	CCS angina class	0	61.5
Kastrup 2011 NOVA [28]	RCT (double-blind)	17	Patients with > 10% reversible ischaemia of LV at an adenosine stress SPECT, a coronary arteriography demonstrating at ≥ 1 main coronary vessel from which new collaterals/vessels could be supplied, CCS ≥ 2 despite optimal medicinal therapy, two baseline bicycle ETTs (> 2 and < 8 min until angina level 3 and exercise duration on the two ETTs should be within 15% of each other)	62 76%	Intramyocardial injection of Ad_GV_VEGF121.10NH	Placebo	Bicycle	Exercise time	Exercise time, CCS angina class, anginal episodes, and NTG use	NR	52.3
Leon [20]	RCT (double-blind)	298	History of coronary artery disease with refractory angina (CCS class III or IV), despite optimal medical therapy	62.9 77%	Direct Myocardial Revascularisation	Sham	Modified Bruce protocol	Exercise time	Exercise time, SAQ	NR	49.3
Losordo [18]	RCT (double-blind)	19	Patients with CCS class III or IV angina refractory to maximum medical therapy, multivessel coronary artery disease not suitable for bypass surgery or angioplasty, and reversible ischemia on stress SPECT Tc 99 m sestamibi nuclear imaging	61 79%	Intramyocardial injections of plasmid DNA encoding for phVEGF2	Saline injection	Modified Bruce protocol	CCS angina class and exercise time	CCS angina class, exercise time, anginal episodes, NTG use	NR	48.8
Losordo [23]	RCT (double-blind)	24	Patients with CCS class III or IV angina to have attempted “best” medical therapy without control of symptoms (taking ≥ 2 antianginals), noncandidates for revascularisation, have ischaemia on nuclear perfusion imaging, to complete > 1 min but < 6 min of a Bruce protocol, and to experience angina during the baseline exercise test	62.4 79%	Intramyocardial Transplantation of Autologous CD34 + Stem Cells	Saline plus 5% autologous serum	Bruce protocol	Arrhythmia monitoring, anginal episodes, NTG use, exercise time, CCS class, QoL	Exercise time, CCS angina class, SAQ, NTG use, anginal episodes	33	NR
Losordo [27] ACT34-CMI	RCT (double-blind)	167	Patients with CCS class III-IV chronic refractory angina despite optimum medical management and with no suitable revascularisation options	61 87%	Intramyocardial injection of autologous CD34 + cells	Placebo injection	Modified Bruce protocol	Angina frequency	Anginal episodes, Exercise time, CCS angina class, NTG use, SAQ	31	59.8
Povsic 2016 The RENEW [31]	RCT (double-blind)	112	Patients with CCS class III or IV angina, ejection fraction of ≥ 25%; reproducible exercise-limiting angina (between 3 and 10 min on 2 consecutive EETs), minimum of 7 angina episodes per week during a 4-week screening period, were on maximally tolerated medical therapy and had demonstrable ischaemia on stress testing	64 84%	Intramyocardial Autologous CD34 + Cell administration	Placebo injection or no intervention	Modified Bruce protocol	Total exercise time	Exercise time, anginal episodes	26	52.8
Salem 2004 BELIEF [19]	RCT (double-blind)	82	Patients with stable CCS class III or IV angina refractory to maximally tolerated doses of > 2 antianginal medications; evidence of reversible myocardial ischaemia on exercise testing or technetium sestamibi stress myocardial perfusion scanning; and ejection fraction > 25% and wall thickness > 8 mm in the target region for PMLR	66 91%	Percutaneous Myocardial Laser Revascularisation	Sham	Bicycle and treadmill	CCS angina class	Exercise time, SAQ	2	63.5
Tse 2007 PROTECT-CAD [24]	RCT (double-blind)	28	Patients with a history of stable CCS class III or IV angina refractory to medical therapy, with no revascularisation option, able to complete > 3 min but < 10 min of treadmill exercise and 1 or 2 coronary territories of viable ischaemic myocardium	66 75%	Direct endomyocardial implantation of bone marrow cells	Autologous plasma injection	Modified Bruce protocol	Exercise time	Exercise time, CCS angina class	NR	50
Van Ramshorst [25]	RCT (double-blind)	50	Patients with severe angina (CCS class III-V) despite optimal medical therapy, and myocardial ischaemia in at least 1 myocardial segment on Tc-99 m tetrofosmin SPECT	64 86%	Intramyocardial Bone Marrow Cell Injection	Placebo solution	Bicycle	Summed stress score	CCS angina class	NR	55.1
Perin [29]	RCT (double-blind)	20	CCS class II to IV angina or NYHA class II or III heart failure (able to walk on a treadmill) on maximum tolerable medical therapy, ejection fraction ≤ 45%, the presence of a reversible perfusion defect on SPECT, coronary artery disease ineligible for percutaneous or surgical revascularisation	58 85%	Transendocardial injection of autologous aldehyde Dehydrogenase bright stem cells	Placebo solution	Not evaluated	Occurrence of adverse events	CCS angina class	100	34.1
Wang [26]	RCT (double-blind)	112	Patients with diffuse triple vessel disease and CCS class III or IV angina, receiving conventional medical therapy, considered non-candidates for conventional revascularisation, required to have ischaemia on nuclear perfusion imaging, to complete > 1 min and < 6 min of a standard Bruce protocol and to experience angina during the baseline exercise test	57.8 51%	Intracoronary Autologous CD34 + Stem Cell Therapy	Placebo solution	Bruce protocol	Safety, angina frequency, NTG use, exercise time, CCS class, SPECT perfusion imaging	Anginal episodes, NTG use, exercise time, CCS class	6	NR
Verheye 2015 COSIRA [30]	RCT (double-blind)	104	CCS class III or IV angina despite efforts to control symptoms with medical therapy for at least 30 days before screening	67.8 81%	Coronary sinus reducer	Sham	Bicycle ergometry stress test (adapted ACIP protocol)	Improvement of 2 or more CCS angina classes	Exercise time, Improvement in CCS class, SAQ	NR	54.2

ACIP, asymptomatic cardiac ischaemia pilot; CCS, Canadian cardiovascular society; EET, exercise tolerance test; HF, heart failure; LV, left ventricle; LVEF, left ventricle ejection fraction; NR, not reported; NTG, nitroglycerine; NYHA, New York heart association; PCI, percutaneous coronary intervention; PMLR, percutaneous myocardial laser revascularisation; RCT, randomised clinical trial; rEF, reduced ejection fraction; SAQ, Seattle angina questionnaire; SPECT, single-photon emission computerised tomography

In eleven studies, the intervention was an intramyocardial injection [18, 21–29, 31] (four containing plasmids [18, 21, 22, 28], four containing autologous CD34 + cells [23, 26, 27, 31], two containing bone marrow cells [24, 25] and one containing autologous aldehyde dehydrogenase bright stem cells [29]) and all had a sham procedure as the control arm. The remaining five studies evaluated other interventions such as PCI (ORBITA trial [5]), coronary sinus reducer (COSIRA trial [30]), myocardial laser revascularisation (Salem [19], Leon [20]), and internal mammary artery ligation (Cobb [17]) versus a sham comparator.

Regarding the sham comparators, seven studies used a placebo solution [22, 25–29, 31], five studies used a sham intervention [5, 17, 19, 20, 30], two studies injected a saline solution in the sham intervention arm [18, 23], one study used a placebo plasmid [21], and one study injected autologous plasma [24].

### Risk of bias

In the risk of bias assessment most of the studies (11 of 16) had moderate (‘some concerns’) risk of bias. Most of these studies were considered to have risk of selection bias of the reported outcomes. Due to the nature of our question/review, this selection is unavoidable because we are searching for the best response in the sham arm. Additionally, we judged three of the sixteen RCTs to have further risks of bias: one due to not having a pre-specified analysis plan; one due to not having a pre-specified analysis plan, and for having baseline imbalances (medication usage) between groups [22]; and one due to lack of information about the allocation sequence and its concealment [20] (Additional file 1).

### Exercise time

The sham intervention arm of thirteen RCTs [5, 17–20, 22–24, 26–28, 30, 31] (n = 13) contributed data for exercise time as the primary outcome. This outcome was measured in six studies with the Treadmill Bruce protocol [5, 17, 19, 22, 23, 26], in five studies with the Treadmill modified Bruce protocol [18, 20, 24, 27, 31], and in two studies with the bicycle protocol (28, 30). Although the study by Fuchs et al. (2006)[22] used the asymptomatic cardiac ischaemia pilot (ACIP) protocol, we considered it to be very similar to the Bruce treadmill protocol and, therefore, we included it in the Treadmill Bruce subgroup. Two of the sixteen included studies did not report data for this outcome [21, 29], and in another study [25] that used a bicycle protocol, the exercise outcome was measured in watts, not in time. We were not able to include two studies [23, 27] for the relative measurement of change since these studies did not present the pre and post-sham values, only mean differences.

**Fig. 2:**
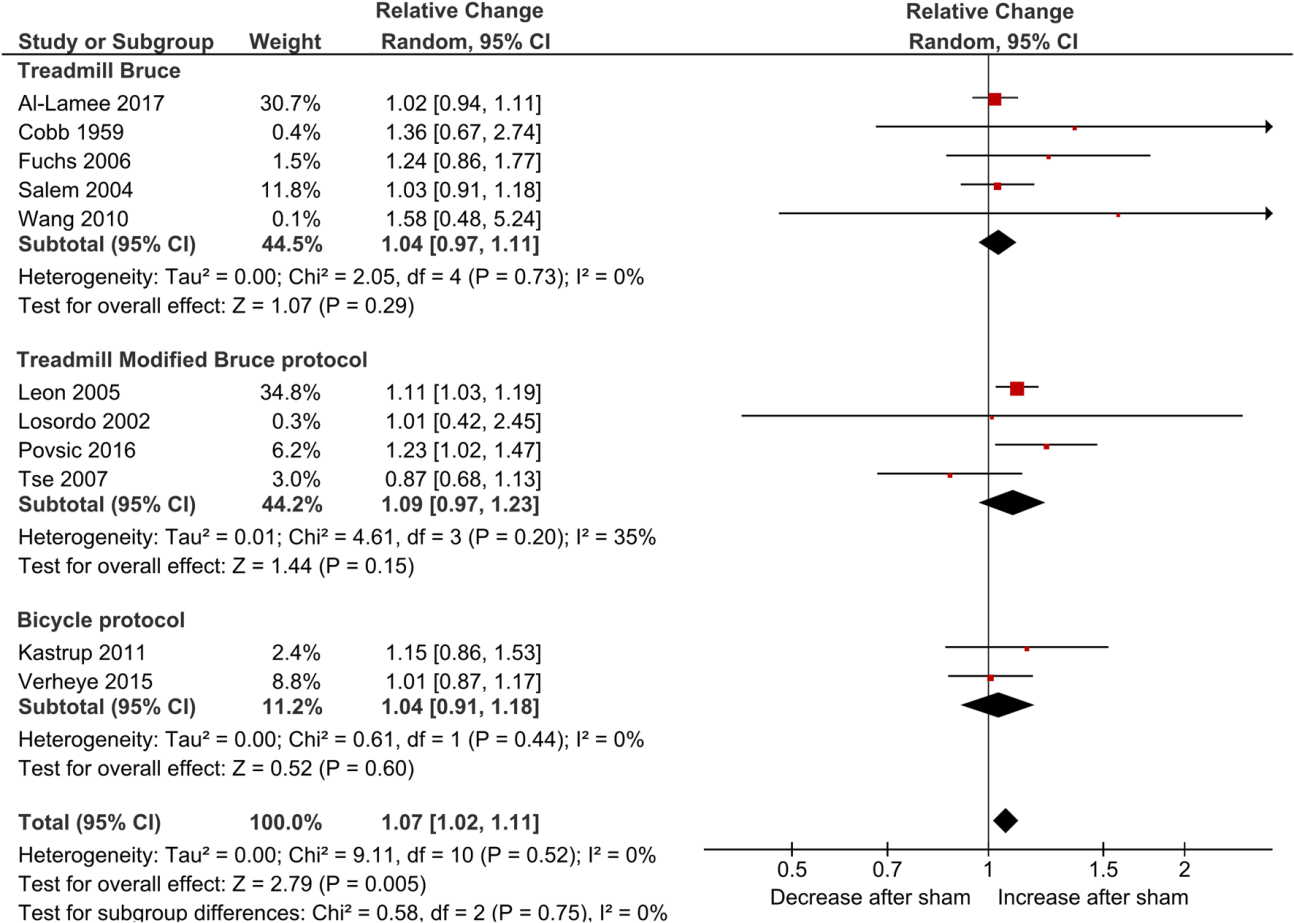
Forest plot for relative change of exercise time

**Fig. 3:**
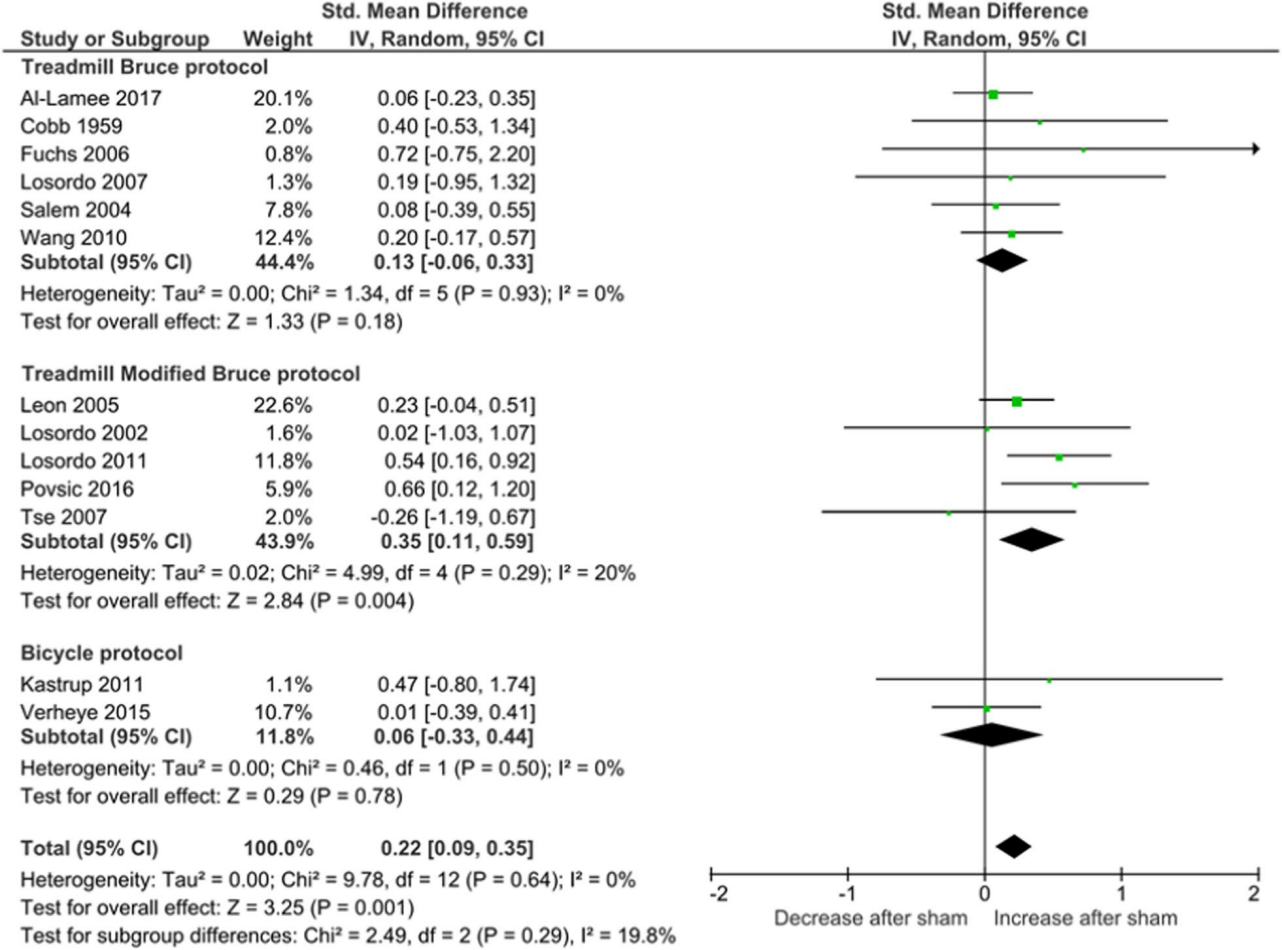
Forest plot for standardised mean exercise difference

Pooled estimates showed an overall significant increase in exercise time with sham, corresponding to 7% using the relative change measure (RC 1.07; 95% CI 1.02–1.11; I^2^ = 0%; Fig. 2) and a Cohen d of 0.22 (SMD 0.22; 95% CI 0.09–0.35; I^2^ = 0%; Fig. 3). There were no differences between subgroups defined according to the way this outcome was measured.

In absolute terms, the sham intervention significantly increased the exercise time as measured by the modified Bruce protocol (MD 56.03; 95% CI 26.92–85.14, I^2^ = 0%), but not when measured by the Bruce (MD 27.10; 95% CI − 12.99 to 67.19, I^2^ = 0%) or bicycle exercise protocols (MD 23.04; 95% CI − 51.07 to 97.16; I^2^ = 0%) (Additional file 2).

### CCS class, frequency of anginal episodes and nitroglycerine use

Overall, ten RCTs [18, 21–26, 28–30], six RCTs [18, 23, 26–28, 31], and five RCTs [18, 23, 26–28] contributed data for the outcomes CCS angina class, anginal episodes per week, and nitroglycerine use, respectively.

In absolute terms, there was a mean decrease in CCS angina class (MD − 0.78; 95% CI − 1.10 to − 0.47; I^2^ = 75%; Additional file 2).

**Fig. 4:**
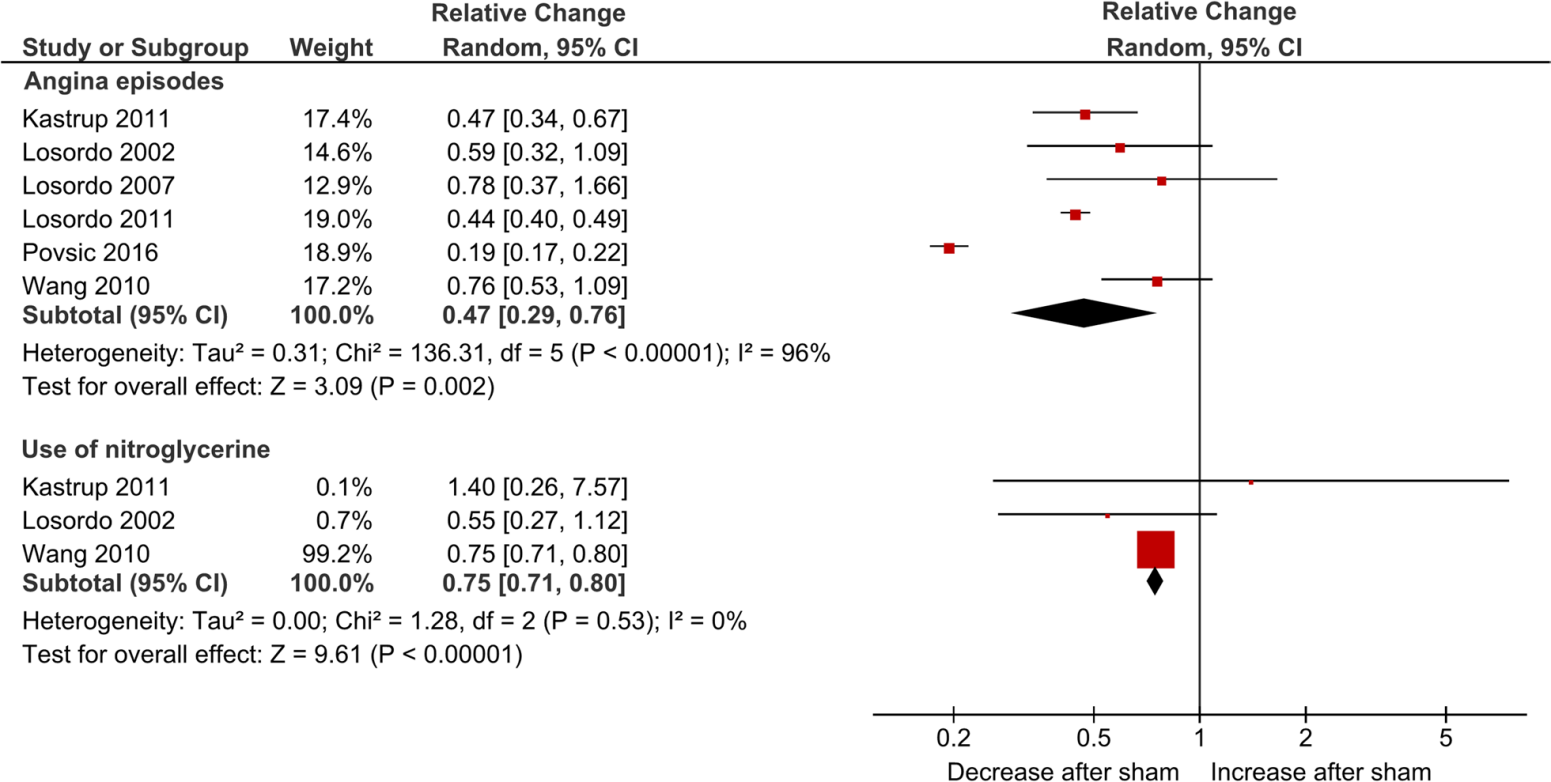
Forest plot for the relative change regarding anginal episodes and nitroglycerine use per week

Sham procedures were associated with a mean 53% and 25% relative decrease in anginal episodes (RC 0.47; 95% CI 0.29–0.76; I^2^ = 96%; Fig. 4) and NTG use (RC 0.75; 95% CI 0.71–0.80; I^2^ = 0%; Fig. 4), respectively.

In absolute terms, there was a significant decrease in anginal episodes per week (MD − 10.29; 95% CI − 13.04 to − 7.54; I^2^ = 0%; supplementary material). The weekly use of NTG was significantly decreased after sham intervention (MD − 3.98; 95% CI − 5.14 to − 2.82; I^2^ = 0%; Additional file 2).

### Quality of life—Seattle Angina Questionnaire

Overall, seven RCTs [5, 19, 20, 22, 23, 27, 30] contributed data for pre-post evaluation of QoL using SAQ within the sham arm of an RCT.

**Fig. 5:**
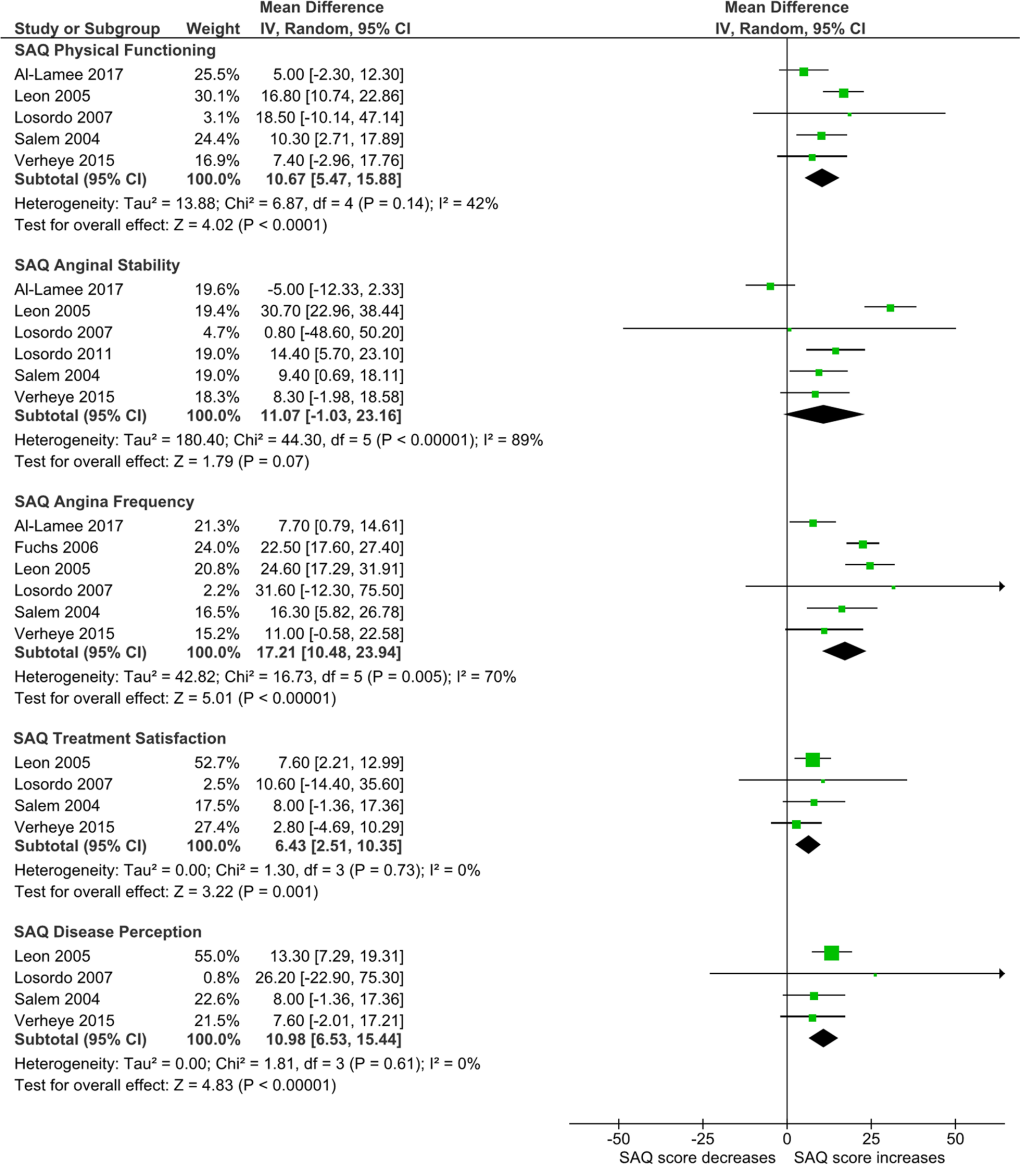
Forest plot for Seattle Angina Questionnaire (SAQ). Subgroups: Physical Limitation, Angina Stability, Angina Frequency, Treatment Satisfaction, and Disease Perception

The sham intervention significantly improved SAQ scores regarding physical functioning (MD 10.67; 95% CI 5.47–15.88; I^2^ = 42%), angina frequency (MD 17.21; 95% CI 10.48–23.94; I^2^ = 70%;), treatment satisfaction (MD 6.43; 95% CI 2.51–10.35; I^2^ = 0%), and disease perception (MD 10.98; 95% CI 6.53–15.44; I^2^ = 0%) (Fig. 5).

### Publication bias risk assessment

The visual interpretation of the funnel plots (Additional file 3) does not suggest the existence of small-study effects or publications bias for all outcomes. A similar interpretation was supported by the results of the Egger’s test (p-value > 0.10) (Additional file 3). In addition, the trim-fill analyses of all outcomes did not substantially change the outcome measures.

### Subgroup/exploratory analyses and heterogeneity investigation

We performed an analysis of relative change in exercise time according to the type of sham intervention: pure sham intervention (RC 1.05; 95%CI 1.01–1.11; I^2^ = 0%) or sham intervention plus placebo solution (RC 1.12; 95%CI 0.98–1.27; I^2^ = 6%), and we did not find any differences between the two subgroups (p-value for interaction 0.42) (Additional file 4).

The subgroup analysis of relative change in exercise time according to the mean reported LVEF showed no difference (p-value for subgroup interaction 0.24) among LVEF < 50% (RC 1.11, 95%CI 1.02–1.19; 2 studies) and LVEF ≥ 50% or LVEF not reported (RC 1.04, 95%CI 0.99–1.10) (Additional file 4).

We also performed meta-regression of relative change in exercise time with age, the proportions of men and diabetic patients. The meta-regression showed that for each year of increase in average population age a relative decrease of 2.3% can be expected in exercise time (− 2.3%, 95% CI − 4.5 to − 1%). The results of meta-regression for men and diabetic patients’ proportions were not significant (Additional file 4).

The measures of exercise time change, both as a relative change (primary outcome) and as SMD (Cohen d) did not have substantial heterogeneity. The analyses that had important statistical heterogeneity were angina episodes frequency (I^2^ = 95%), CCS class analysis (I^2^ = 70%), and nitroglycerine use (I^2^ = 95%). The investigation showed that estimates of the subgroup of patients with lower LVEF/HF patients (RC 0.66, 95%CI 0.41–1.06; two studies) did not have substantial heterogeneity (I^2^ = 0% for angina episodes and CCS class, I^2^ = 46% for nitroglycerine use) and the reduction in the CCS class was still statistically significant, but not different from LVEF > 50% (p-value for interaction 0.21).

For SAQ outcomes, angina stability (I^2^ = 89%) and angina frequency (I^2^ = 70%) showed substantial heterogeneity. Similar to previous outcomes, heterogeneity was reduced in the subgroup of patients with lower LVEF/HF (27% for SAQ angina stability and 0% for SAQ angina frequency). SAQ angina stability did not show substantial heterogeneity in the subgroup sham intervention plus placebo solution.

The exclusion of studies at higher risk of bias only improved the heterogeneity of SAQ angina frequency (I^2^ = 0%).

### Certainty of evidence for exercise time using GRADE

We evaluated the certainty of the pooled evidence for the exercise time using the GRADE framework. The certainty about evidence was low due to the pre-post evaluation of outcomes in the sham arm and due to the selective reporting bias risk inherent to search of the best response within trial as an equivalent of a sham/placebo effect. The GRADE table is depicted in Table 2.

**Table 2:** GRADE assessment of pooled evidence

Outcome	GRADE certainty assessment							Effect (95% CI)
	Participants (studies)	Risk of bias	Inconsistency	Indirectness	Imprecision	Publication bias	Overall certainty of evidence	
Exercise time ratio	393* (11 sham arms of RCTs)	Very Serious**	not serious	not serious	not serious	none	⨁⨁◯◯ Low	RR 1.07 (1.02–1.11)
Exercise time in SMD	454 (13 sham arms of RCTs)	Very Serious**	not serious	not serious	not serious	none	⨁⨁◯◯ Low	SMD 0.22 higher (0.09 higher to 0.35 higher)

CI: confidence interval; RC: relative change (similar to risk ratio); SMD: standardised mean difference; *Two studies did not report baseline data to calculate the exercise time ratio (only provided data about change). ** Data derived from pre-post effect in the sham arm of RCTs, and high risk of selective reporting of the outcome measure

## Discussion

This systematic review and meta-analysis showed that a sham intervention in chronic coronary syndromes can increase the exercise time in a stress test by 7%, halve the number of episodes of angina, improve CCS class, and reduce nitroglycerine in 25% of patients. The sham intervention also improved domains covered by the SAQ, namely physical functioning, angina frequency, treatment satisfaction, and disease perception.

The average increase in exercise for the stress test was 7% but different types of stress tests were included in this outcome making it difficult to ascertain the clinical significance of this effect. To overcome this limitation, we also used the Cohen D (SMD) and the pooled analysis sought a statistically significant increase of SMD in exercise trials by 0.22, which is deemed to be a change of small magnitude.

In our systematic review, we determined that, for the modified Bruce treadmill protocol, the pooled effect was 56 s (27 s for the Bruce protocol and 23 s for bicycle protocols). One of the included studies, Leon [20], considered that 1 min was the minimal clinically relevant difference in exercise time in the modified Bruce protocol, when comparing an intervention with a sham comparator. This means that minimal clinically relevant difference, in this case, would double the sham effect. Nevertheless, it is worth noting that the sham and Hawthorne effects in this context were able to improve exercise time, which is a relevant prognostic factor in patients with chronic coronary syndromes [32], but no referral should be made on this basis. Despite these considerations, and in the presence of negative results in most invasive studies, our data should be used to emphasise the importance of sham-controlled studies and to perform sample size calculations based on our assumptions. There are some examples, namely in ablation procedures for renal denervation in arterial hypertension [33, 34] that show that it might be worth performing sham-controlled trials to assess the ‘real’ value of the intervention.

Comparing our results with the placebo effect in placebo-controlled trials (where ranolazine and ivabradine were tested) [35, 36] using the same methods, the placebo effect revealed heterogeneous increases between 1.5% and 15%. These results are not very far from the 95% confidence intervals and the factors associated with placebo effects might be similar to those associated with sham effects.

The sham effect was also substantial in decreasing anginal episodes, CCS class, and nitroglycerine consumption as a surrogate to angina severity. The most impressive estimate in this set of outcomes relies on the halving of angina episodes. The most important drawback of this result concerns statistical heterogeneity. However, it is worth noting that when we considered only the two studies with lower left ventricular ejection fraction/higher proportion of heart failure patients, the results did not yield statistical heterogeneity and still showed a significant reduction in anginal episodes of 44%. The CCS class mean reduction was 0.78, which means that most patients improved by at least one class. A decrease of just one class could mean that a patient could walk a longer distance and perform more activities, which correlates with a better QoL. In light of these data, the reduction in nitroglycerine consumption was expected. This can be interpreted as a severity marker of angina but this threshold to treatment with nitroglycerine is an outcome that can be influenced by expectations and thus responsive to the sham effect.

Most of the domains of the SAQ, except for angina stability, were significantly improved after sham interventions. The relevance of our results once again emphasises the need for sham-controlled trials to interpret the outcomes. For example, an open-label trial showed that percutaneous coronary revascularisation compared with optimal medical treatment significantly improved SAQ in the domains of physical limitation, angina frequency, and disease perception by 5.2, 5.2, and 6.6 points respectively [37]. A pre-post evaluation of percutaneous coronary revascularisation in this context also showed an increase of 13.1 points in physical limitation, 14.6 in angina frequency, and 21.3 for disease perception/QoL. The results of our systematic review showed that sham interventions can increase these SAQ domains to 10.67, 17.21, and 10.98, respectively. All the significant comparisons with the optimal medical treatment had a magnitude lower than the sham effect, and within the intervention, the SAQ physical limitation and angina frequency had results within the 95% confidence intervals of the sham effects. We are not claiming that the intervention has no additional therapeutic value, but conclude that any fair evaluation (even for the intervention in case of efficacy) would require a sham arm for adequate interpretation, however, we recognise the additional challenges in performing such studies.

Overall, these results showed that patients with chronic coronary syndromes improve after a sham invasive procedure. This seems to call into question the meaning of the relief reported by patients with angina submitted to invasive interventions and requires that we re-think the role of placebo in contributing to this relief. In cardiovascular medicine there are examples of treatments considered clearly beneficial where an initial placebo-controlled trial was rejected, before, years later, an exploratory/pragmatic trial (such as ORBITA) shakes the validity of some of these principles and triple-blinded (patient, physician, outcome assessor) placebo/sham-controlled trials are valued and required [5].

Current recommendations point to revascularisation as having a central role in the management of CCS when angina persists despite treatment with an antianginal drug, but also recommend that individual benefit-risk ratio be considered and that decision-making be shared with the patient [1], keeping in mind comparisons of an intervention to a sham intervention or placebo, while integrating the values and preferences of the patient, for example, their aversion to invasive treatment.

The results of this systematic review also highlight the need for randomised controlled trials to evaluate the efficacy of invasive treatments in patients with CCS and establish, for the measured outcomes, an assumed effect that should account for this sham effect in future trials. Also, the efficacy of blinding, for both patients and investigators, should be considered a mandatory outcome to report in these trials. In the ORBITA trial, the authors reported a blinding index and raw data of arm guessing. The blinding index was significant in the PCI arm, but raw data show that 47.6% of PCI patients guessed their allocation arm correctly, 28.6% were wrong and the remainder did not know. In the placebo/sham arm, only 37.4% guessed their arm.

### Limitations

The results and conclusions of this systematic review must account for the limitations of the individual studies included here. Most of the studies presented their results as study-level data and not as individual patient data, which can lead to biased assessments and limited interpretation of the data. In addition, eleven of the studies were judged as having a risk of bias.

We were also limited by the variety of interventions and types of sham in published studies, which is also partially responsible for the heterogeneous population. Another limitation concerns the standardised mean difference analyses of exercise time, which reported only a small change, diminishing the robustness of our results. Besides, most of our outcomes are subjective patient-reported outcomes which makes them prone to being modulated by a putative placebo/sham effect.

Furthermore, in clinical practice, therapeutic adjustments are made, according to the individual patient’s necessities and these adjustments can alter the manifestations of the disease, possibly resulting in an improvement of symptoms or other disease classification parameters. Throughout the follow-up periods of the selected trials, which varied from 6 weeks to 12 months, medication adjustments could have been undertaken, affecting the results of the measured outcomes, which cannot be accounted for or adjusted. We also considered the follow-up periods to be short-term observation times, which prevented us from drawing conclusions about the effect over a longer period.

Our results could be affected, not only by the therapeutic adjustments, but also by the natural progression of the disease and (its) regression to the mean since it is expected that the invasive treatments are proposed for the most symptomatic patients.

Finally, we were limited by the lack of studies presenting another control group, with no intervention and no blind placebo.

## Conclusions

This systematic review suggests that sham invasive interventions in patients with chronic coronary syndromes are associated with a significant decrease in anginal episodes per week, NTG use per week and CCS angina class, and increases in exercise time and SAQ QoL scores. These results indicate we need to be cautious when interpreting previous clinical trials that are not placebo-controlled, and reinforce the importance of evaluating the efficacy of new invasive treatments against a placebo procedure.

## Supplementary Information

**Additional file 1:** Search strategy, study selection and risk of bias.

**Additional file 2:** Analyses of exercise time (measured as mean difference, and subgroup analyses), CCS class change and anginal episodes.

**Additional file 3:** Publication bias.

**Additional file 4:** Sensitivity analyses and meta-regressions.

## Data Availability

All data generated or analysed during this study are included in this published article and its Additional files.

## Abbreviations

ACIP: Asymptomatic cardiac ischaemia pilot
CAD: Coronary artery disease
CCS: Canadian cardiovascular society
CI: Confidence interval
EET: Exercise tolerance test
HF: Heart failure
LV: Left ventricle
LVEF: Left ventricle ejection fraction
MD: Mean difference
NTG: Nitroglycerine
NYHA: New York heart association
PCI: Percutaneous coronary intervention
PMLR: Percutaneous myocardial laser revascularisation
QoL: Quality of life
RC: Relative change
RCT: Randomised clinical trial
rEF: Reduced ejection fraction
SAQ: Seattle angina questionnaire
SD: Standard deviation
SMD: Standardised mean difference
SPECT: Single-photon emission computerised tomography
