# Supplementary material for: The sham effect of invasive interventions in chronic coronary syndromes: a systematic review and meta-analysis
Source: BMC Cardiovasc Disord. 2022 May 14;22:223. doi: 10.1186/s12872-022-02658-x (PMC9107755; doi:10.1186/s12872-022-02658-x)
Supplement: Supplementary file 1 — Additional file 1: Search strategy, study selection and risk of bias. [file 12872_2022_2658_MOESM1_ESM.pdf]

ADDITIONAL FILE 1

SEARCH, STUDY SELECTION AND RISK OF BIAS

The sham effect of invasive interventions in chronic coronary syndromes – a systematic review and meta-analysis

Authors: Catarina Palma, Cláudio David MD, Ricardo M Fernandes MD PhD, Fausto J Pinto MD PhD, João Costa MD PhD, Joaquim J Ferreira MD PhD, Daniel Caldeira MD PhD

Table of contents

Search Strategy 4
Supplementary Tables 5
Supplementary Table 1: Reasons for exclusion in full-text review 5
Supplementary Figures 8
Supplementary Figure 1: Risk of Bias of included studies 8

Search Strategy

#	Searches
1	exp Angina Pectoris/
2	exp Angina, Stable/
3	angina.ti,ab.
4	coronary.ti,ab.
5	exp Myocardial Ischemia/
6	exp Coronary Artery Disease/
7	1 or 2 or 3 or 4 or 5 or 6
8	random*.af.
9	sham.ti,ab.
10	sham*.ti,ab.
11	blind*.ti,ab.
12	8 or 11
13	9 or 10
14	7 and 12 and 13
15	exp animals/ not humans.sh.
16	14 not 15
17	remove duplicates from 16

Supplementary Tables

Supplementary Table 1: Reasons for exclusion in full-text review

Amin 2010	Non-invasive intervention – the study uses Enhanced External Counterpulsation, which is considered a non-invasive therapy.
Arora 1999	Non-invasive intervention – the study uses Enhanced External Counterpulsation, which is considered a non-invasive therapy.
Arora 2002	Non-invasive intervention – the study uses Enhanced External Counterpulsation, which is considered a non-invasive therapy.
Avery 2014	Non-invasive intervention – the study uses Enhanced External Counterpulsation, which is considered a non-invasive therapy.
Ballegaard 1986	Non-invasive intervention – the study uses acupuncture, which is considered a non-invasive therapy.
Ballegaard 1990	Non-invasive intervention – the study uses acupuncture, which is considered a non-invasive therapy.
Ballegaard 1991	Non-invasive intervention – the study uses acupuncture, which is considered a non-invasive therapy.
Banai 2013	Not a RCT – this study is an open-label trial, not a randomized controlled trial.
Bartunek 2017	No data – this study does not provide outcomes of interest.
Beck 2015	Non-invasive intervention – the study uses Enhanced External Counterpulsation, which is considered a non-invasive therapy.
Beres 2006	No sham procedure – this study does not include a sham procedure.
Brockow 2000	Not a RCT – this study is a review.
Casey 2011	Non-invasive intervention – the study uses Enhanced External Counterpulsation, which is considered a non-invasive therapy.
Corcoran 2018	Not a RCT – this study is the definition of a study design, without results.
Dimond 1960	No data – the study does not provide enough information about the sham group and its results.
Eddicks 2007	No data – the study does not provide information about the group that starts with the sham intervention.
Fisher 2013	Not a RCT – this study is a review.
Glazachev 2018	Non-invasive intervention – the study uses Intermittent hypoxia-hyperoxia, which is considered a non-invasive therapy.

Hausenloy 2016	Non-invasive intervention – the study uses Remote isquemic preconditioning, which is considered a non-invasive therapy.
Hautvast 1998	Unblinded – this study is not patient blinded.
Hoffmann 2015	Not a RCT – this study is the definition of a study design, without results.
Jeong 2013	Not a RCT – this study is a review.
Jimenez-Quevedo 2014	No sham procedure – this study does not include a sham procedure.
Jolicoeur 2013	Not a RCT – this study is the definition of a study design, without results.
Kandala 2013	Not a RCT – this study is a review.
Khan 2014	Not a RCT – this study is a case report.
Khan 2015	Not a RCT – this study is the definition of a study design, without results.
Khan 2015	Not a RCT – this study is the definition of a study design, without results.
Khan 2017	No data – this study is a crossover randomized controlled trial, with a sham arm. The results are presented for the overall period of the sham intervention, in both groups, not individualized results before and after the crossover.
Kikuchi 2010	Non-invasive intervention – the study uses cardiac shock wave therapy, which is considered non-invasive.
Konigstein 2014	No sham procedure – this study does not include a sham procedure.
Kuehn 2018	Not a RCT – the study is a narrative review.
Lanza 2011	No sham procedure – this study uses a low stimulation group, not considered a true sham procedure.
Lanza 2012	Not a RCT – the study is an abstract.
Leibowitz 2012	Non-invasive intervention – the study uses cardiac shock wave therapy, which is considered non-invasive.
Miller 2012	Not a RCT – this study is a review.
Nnoaham 2008	Not a RCT – this study is a review.

Perin 2012	No data - the study does not provide information about some additional explored outcomes.
Pokushalov 2009	No sham procedure – this study does not include a sham procedure.
Salem 2006	No sham procedure – this study does not include a sham procedure.
Saririan 2003	Not a RCT – this study is a review.
Shkolnik 2018	Non-invasive intervention – the study uses cardiac shock wave therapy, which is considered non-invasive.
Shkolnik 2018	Not a RCT – this study is the definition of a study design, without results.
Soran 1999	Not a RCT – this study is a review.
Szatkowski 2002	Not a RCT – this study is a review.
Thadani 2014	Not a RCT – this study is a review.
Tsivgoulis 2017	Not a RCT – this study is a review.
Van Ramshorst 2011	No data - the study does not provide information about our selected outcomes.
Zipes 2012	No sham procedure – this study uses a low stimulation group, not considered a true sham procedure.

Supplementary Figures

	Randomization process	Deviations from intended interventions	Missing outcome data	Measurement of the outcome	Selection of the reported result	Overall
Al-lamee2017	+	+	+	+	+	+
Cobb1959	+	+	+	+	+	+
Fuchs2006	!	+	+	+	!	!
Kastrup2005	+	+	+	+	!	!
Kastrup2011	+	+	+	+	!	!
Leon2005	!	+	+	+	!	!
Losordo2002	+	+	+	+	+	+
Losordo2007	+	+	+	+	!	!*
Losordo2011	+	+	+	+	!	!
Salem2004	+	+	+	+	!	!
Tse2007	+	+	+	+	+	+
Ramshorst2009	+	+	+	+	!	!
Verheye2015	+	+	+	+	+	+
Povsic2016	+	+	+	+	!	!
Wang2010	+	+	+	+	!	!
Perin2012	+	+	+	+	!	!

+ Low risk
! Some concerns
– High risk

*non-powered pilot study in the overall assessment (Losordo 2007)

Supplementary Figure 1: Risk of Bias of included studies
