# Supplementary material for: The sham effect of invasive interventions in chronic coronary syndromes: a systematic review and meta-analysis
Source: BMC Cardiovasc Disord. 2022 May 14;22:223. doi: 10.1186/s12872-022-02658-x (PMC9107755; doi:10.1186/s12872-022-02658-x)
Supplement: Supplementary file 2 — Additional file 2: Analyses of exercise time (measured as mean difference, and subgroup analyses), CCS class change and anginal episodes. [file 12872_2022_2658_MOESM2_ESM.pdf]

ADDITIONAL FILE 2

Analyses of exercise time (measured as mean difference, and subgroup analyses)
CCS class change and anginal episodes

The sham effect of invasive interventions in chronic coronary syndromes – a systematic review and meta-analysis

Authors: Catarina Palma, Cláudio David MD, Ricardo M Fernandes MD PhD, Fausto J Pinto MD PhD, João Costa MD PhD, Joaquim J Ferreira MD PhD, Daniel Caldeira MD PhD

Table of contents

Supplementary Figure: Mean difference of exercise time 3

Supplementary Figure: Analysis of relative change for exercise time according with the type of sham 3

Supplementary Figure: Analysis of relative change for exercise time according with the mean LVEF/proportion of HF patients. HF: Heart failure; NR: not reported 4

Supplementary Figure: Mean difference of Canadian Cardiovascular Society angina class. 4

Supplementary Figure: Mean difference of anginal episodes and nitroglycerine use per week. 5

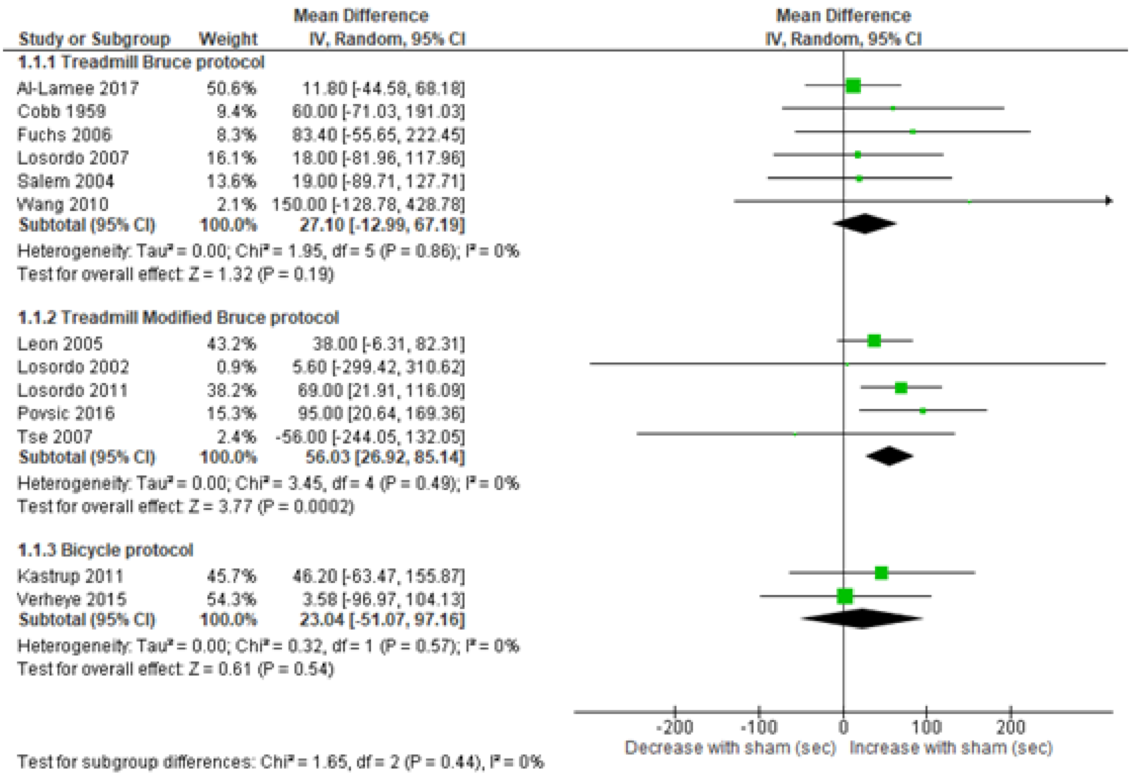

Supplementary Figure: Mean difference of exercise time

Pooled and individual mean difference and 95% CI for the exercise time outcome with Bruce protocol, modified Bruce protocol and bicycle protocol.

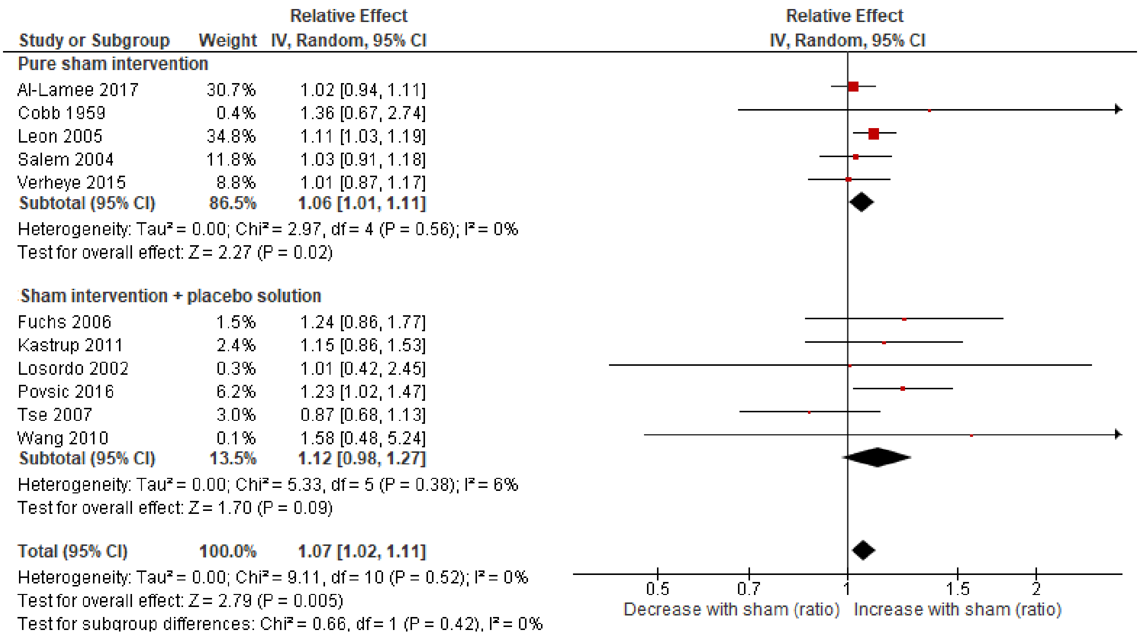

Supplementary Figure: Analysis of relative change for exercise time according with the type of sham

Pooled and individual relative effect and 95% CI for exercise time according with the type of sham (pure sham intervention or sham intervention + placebo solution).

Study or Subgroup	Weight	Risk Ratio IV, Random, 95% CI
**LVEF<50%**		
Leon 2005	34.8%	1.11 [1.03, 1.19]
Losordo 2002	0.3%	1.01 [0.42, 2.45]
**Subtotal (95% CI)**	**35.0%**	**1.11 [1.02, 1.19]**
Heterogeneity: Tau² = 0.00; Chi² = 0.04, df = 1 (P = 0.85); I² = 0%		
Test for overall effect: Z = 2.60 (P = 0.009)		
**LVEF≥50%/NR**		
Al-Lamee 2017	30.7%	1.02 [0.94, 1.11]
Cobb 1959	0.4%	1.36 [0.67, 2.74]
Fuchs 2006	1.5%	1.24 [0.86, 1.77]
Kastrup 2011	2.4%	1.15 [0.86, 1.53]
Povsic 2016	6.2%	1.23 [1.02, 1.47]
Salem 2004	11.8%	1.03 [0.91, 1.18]
Tse 2007	3.0%	0.87 [0.68, 1.13]
Verheye 2015	8.8%	1.01 [0.87, 1.17]
Wang 2010	0.1%	1.58 [0.48, 5.24]
**Subtotal (95% CI)**	**65.0%**	**1.04 [0.99, 1.10]**
Heterogeneity: Tau² = 0.00; Chi² = 7.69, df = 8 (P = 0.46); I² = 0%		
Test for overall effect: Z = 1.56 (P = 0.12)		
**Total (95% CI)**	**100.0%**	**1.07 [1.02, 1.11]**
Heterogeneity: Tau² = 0.00; Chi² = 9.11, df = 10 (P = 0.52); I² = 0%		
Test for overall effect: Z = 2.79 (P = 0.005)		
Test for subgroup differences: Chi² = 1.38, df = 1 (P = 0.24), I² = 27.7%		

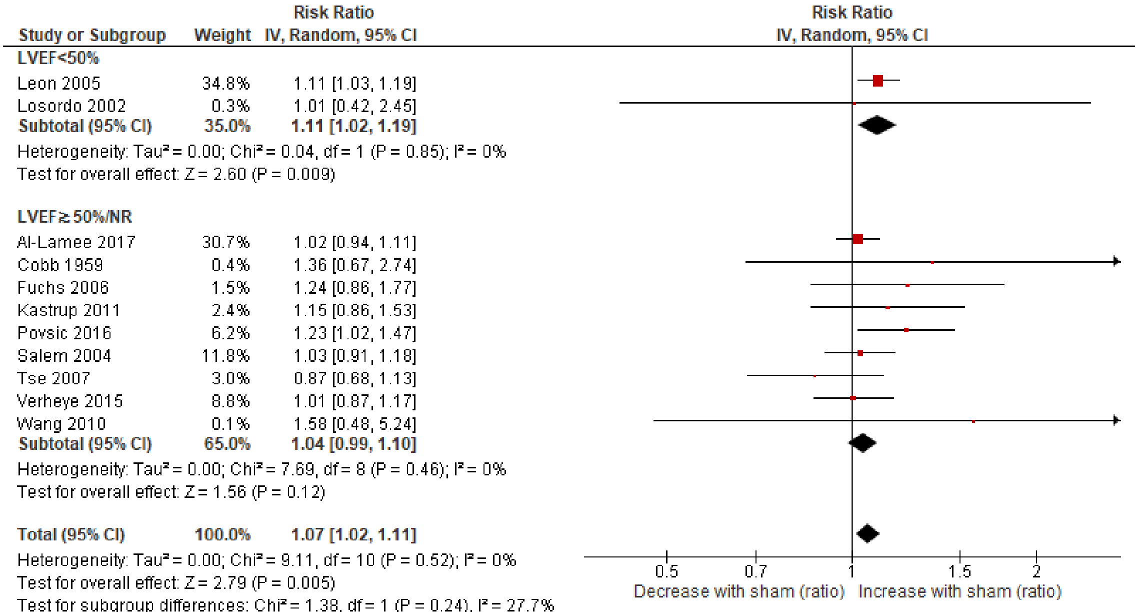

Supplementary Figure: Analysis of relative change for exercise time according with the mean LVEF/proportion of HF patients. HF: Heart failure; NR: not reported

Study or Subgroup	Weight	Mean Difference IV, Random, 95% CI
Fuchs 2006	8.1%	-2.00 [-2.80, -1.20]
Kastrup 2005	16.0%	-1.20 [-1.41, -0.99]
Kastrup 2011	7.1%	-1.00 [-1.90, -0.10]
Losordo 2002	9.3%	-0.20 [-0.90, 0.50]
Losordo 2007	3.9%	-0.80 [-2.20, 0.60]
Perin 2012	12.9%	-0.50 [-0.94, -0.06]
Tse 2007	13.7%	-0.80 [-1.18, -0.42]
van Ramshorst 2009	12.8%	-0.40 [-0.85, 0.05]
Verheye 2015	15.4%	-0.50 [-0.76, -0.24]
Wang 2010	0.8%	-0.80 [-4.26, 2.66]
**Total (95% CI)**	**100.0%**	**-0.78 [-1.10, -0.47]**
Heterogeneity: Tau² = 0.15; Chi² = 35.69, df = 9 (P < 0.0001); I² = 75%		
Test for overall effect: Z = 4.89 (P < 0.00001)		

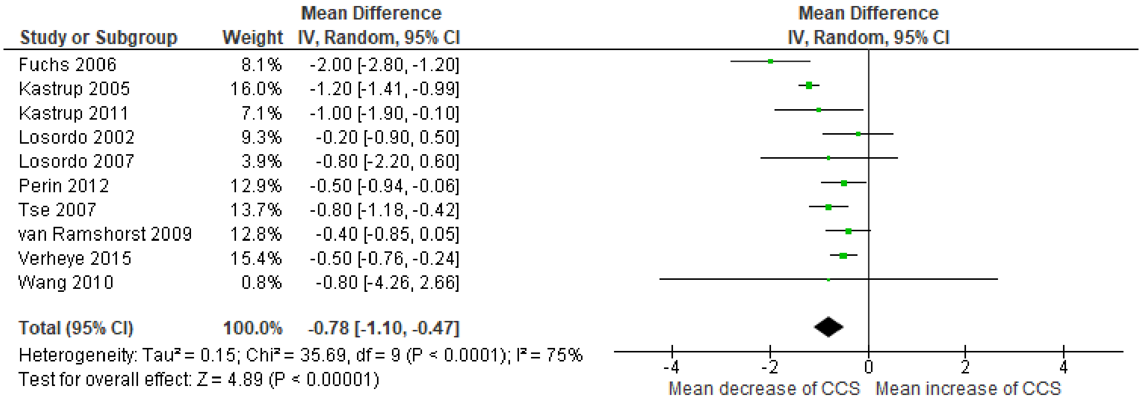

Supplementary Figure: Mean difference of Canadian Cardiovascular Society angina class.

Pooled and individual mean difference and 95% CI for the CCS angina class outcome.

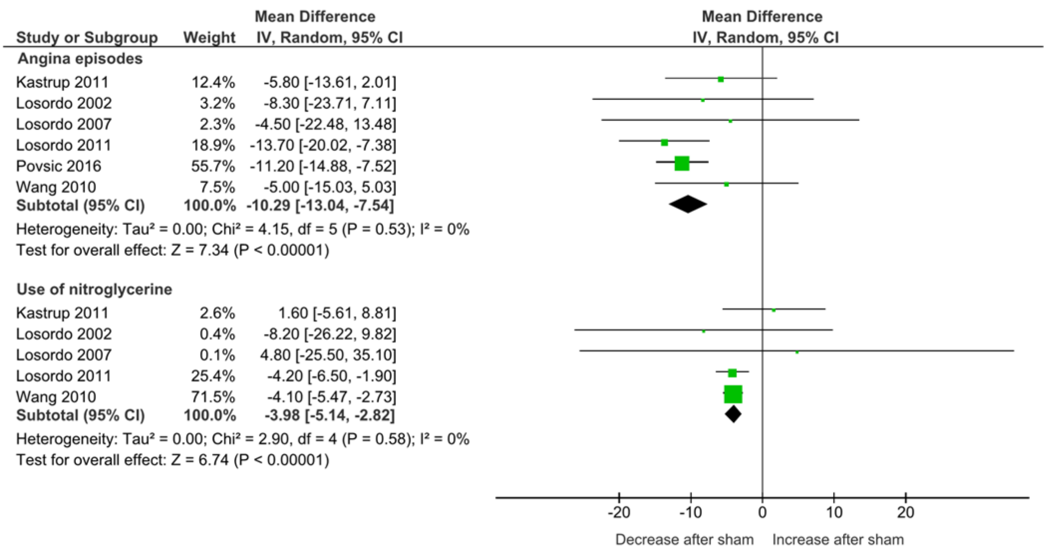

Supplementary Figure: Mean difference of anginal episodes and nitroglycerine use per week.

Pooled and individual mean difference and 95% CI for the anginal episodes and nitroglycerine use per week outcome.
