# Supplementary material for: The sham effect of invasive interventions in chronic coronary syndromes: a systematic review and meta-analysis
Source: BMC Cardiovasc Disord. 2022 May 14;22:223. doi: 10.1186/s12872-022-02658-x (PMC9107755; doi:10.1186/s12872-022-02658-x)
Supplement: Supplementary file 3 — Additional file 3: Publication bias. [file 12872_2022_2658_MOESM3_ESM.pdf]

ADDITIONAL FILE 3

PUBLICATION BIAS

The sham effect of invasive interventions in chronic coronary syndromes – a systematic review and meta-analysis

Authors: Catarina Palma, Cláudio David MD, Ricardo M Fernandes MD PhD, Fausto J Pinto MD PhD, João Costa MD PhD, Joaquim J Ferreira MD PhD, Daniel Caldeira MD PhD

Table of contents

Supplementary Table: Publication/small studies Effect bias evaluation 4

Supplementary Figure: Funnel plot for the analysis of exercise time relative change (upper panel); Trim-fill analyses with the addition of 2 estimates and the resulting forest plot (lower panel).. 5

Supplementary Figure: Funnel plot for the analysis of exercise time SMD (upper panel); Trim-fill analyses with the addition of 1 estimate and the resulting forest plot (lower panel).. 6

Supplementary Figure: Funnel plot for the analysis of absolute mean difference of exercise time (upper panel); Trim-fill analyses with the addition of 1 estimate and the resulting forest plot (lower panel). 7

Supplementary Figure: Funnel plot for the analysis of angina episodes relative change (upper panel); Trim-fill analyses with the addition of 3 estimates and the resulting forest plot (lower panel).. 8

Supplementary Figure 10: Funnel plot for the analysis of absolute mean difference of angina episodes (upper panel); Trim-fill analyses with the addition of 2 estimates and the resulting forest plot (lower panel). 9

Supplementary Figure 11: Funnel plot for the analysis of CCS class; The trim-fill analysis did not add any further study. 10

Supplementary Figure: Funnel plot for the analysis of relative change in NTG use (upper panel); Trim-fill analyses with the addition of 1 estimate and the resulting forest plot (lower panel).. 11

Supplementary Figure: Funnel plot for the analysis of mean difference of NTG use (upper panel); Trim-fill analyses with the addition of 1 estimate and the resulting forest plot (lower panel).. 12

Supplementary Figure: Funnel plot for the analysis of SAQ physical functioning (upper panel); Trim-fill analyses with the addition of 1 estimate and the resulting forest plot (lower panel).. 13

Supplementary Figure: Funnel plot for the analysis of SAQ anginal stability (upper panel); Trim-fill analyses with the addition of 1 estimate and the resulting forest plot (lower panel).. 14

Supplementary Figure: Funnel plot for the analysis of SAQ anginal frequency (upper panel); Trim-fill analyses with the addition of 1 estimate and the resulting forest plot (lower panel).. 15

Supplementary Figure: Funnel plot for the analysis of SAQ treatment satisfaction (upper panel); Trim-fill analyses with the addition of 1 estimate and the resulting forest plot (lower panel).. 16

Supplementary Figure: Funnel plot for the analysis of SAQ disease perception (upper panel); The trim-fill analysis did not add any further study. 17

Supplementary Table: Publication/small studies Effect bias evaluation

Publication/small studies Effect bias evaluation		
Outcome	Outcome subtype	p-value Egger
Exercise Time	Relative change	0.361
	SMD	0.667
	Mean difference	0.802
Angina episodes	Relative change in angina episodes	0.126
	Mean difference for angina episodes	0.209
CCS class	CCS	0.729
NTG use	Relative change in NTG use	0.871
	Mean change in NTG use	0.411
SAQ	Physical functioning	0.859
	Angina stability	0.709
	Angina frequency	0.785
	Treatment satisfaction	0.874
	Disease perception	0.956

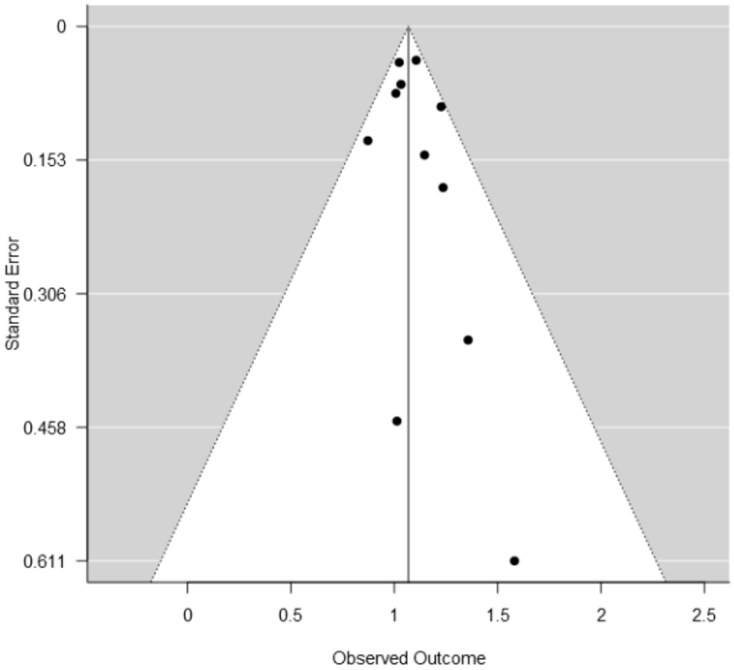

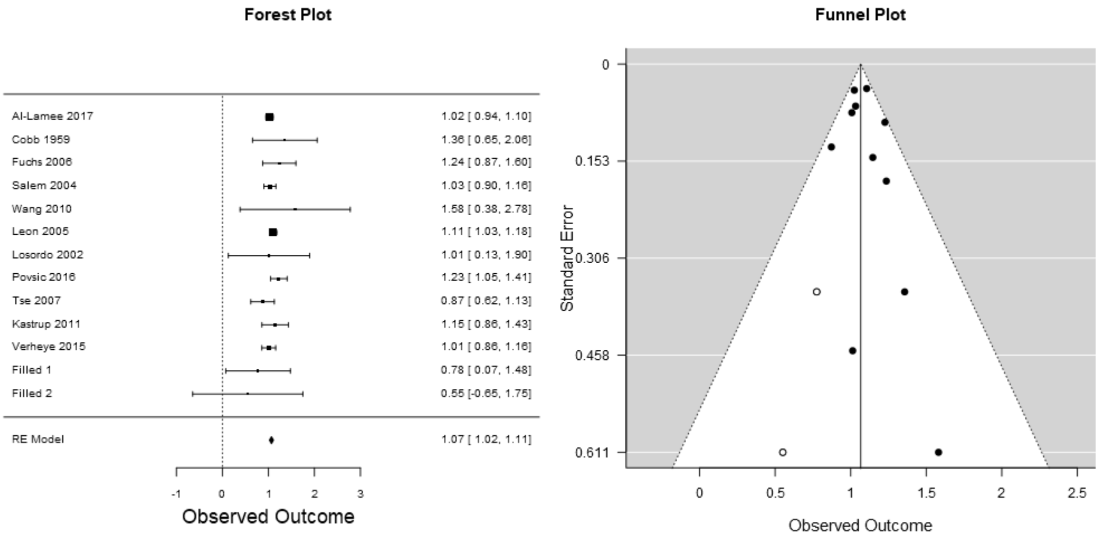

Supplementary Figure: Funnel plot for the analysis of exercise time relative change (upper panel); Trim-fill analyses with the addition of 2 estimates and the resulting forest plot (lower panel).

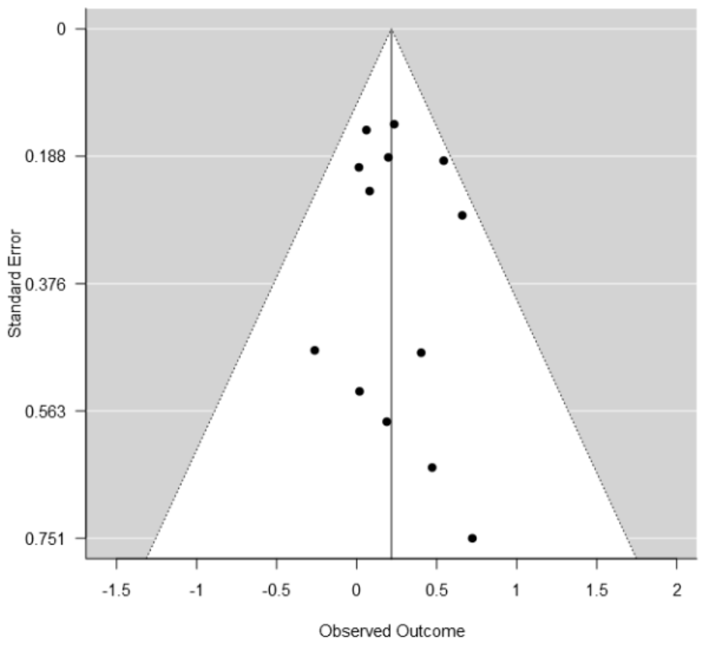

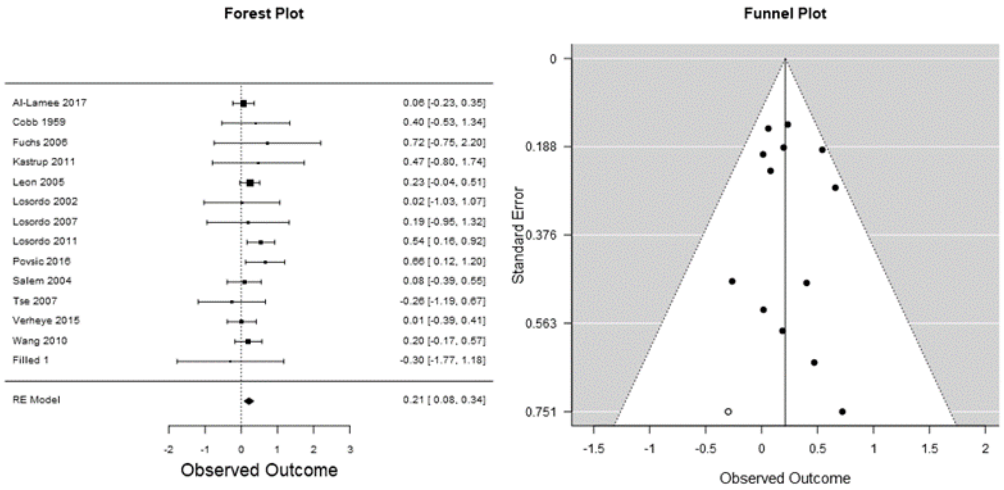

Supplementary Figure: Funnel plot for the analysis of exercise time SMD (upper panel); Trim-fill analyses with the addition of 1 estimate and the resulting forest plot (lower panel).

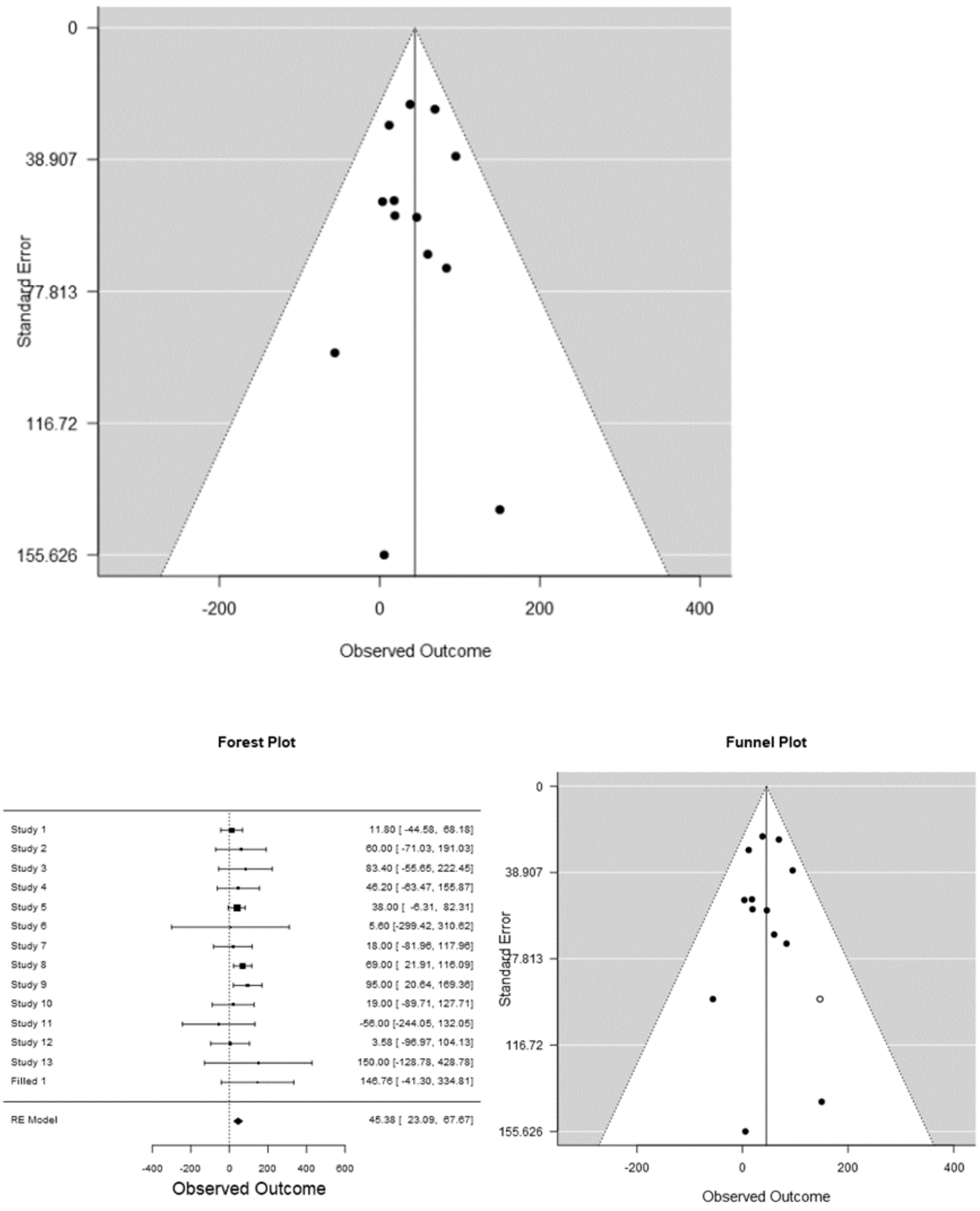

Supplementary Figure: Funnel plot for the analysis of absolute mean difference of exercise time (upper panel); Trim-fill analyses with the addition of 1 estimate and the resulting forest plot (lower panel).

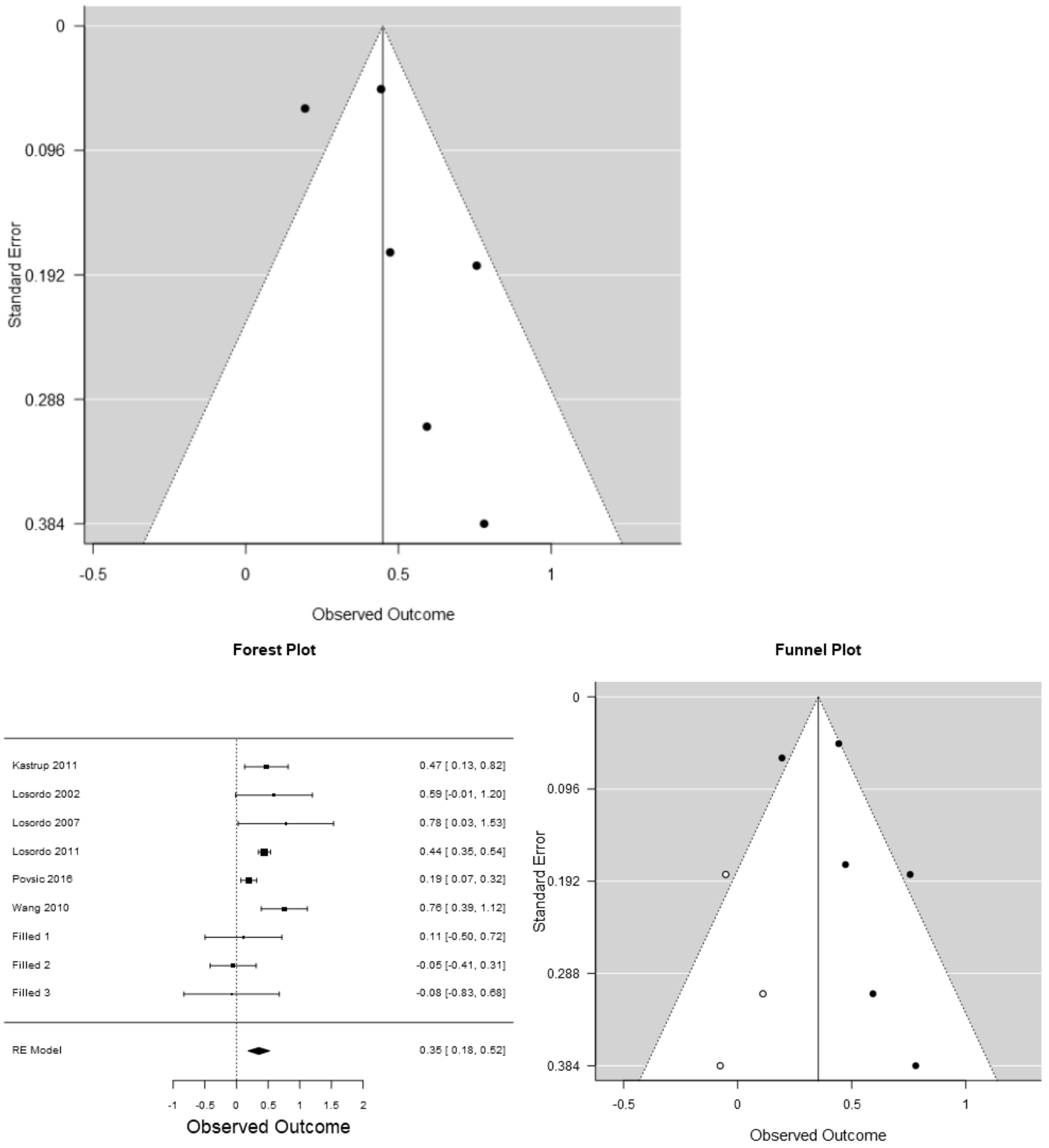

Supplementary Figure: Funnel plot for the analysis of angina episodes relative change (upper panel); Trim-fill analyses with the addition of 3 estimates and the resulting forest plot (lower panel).

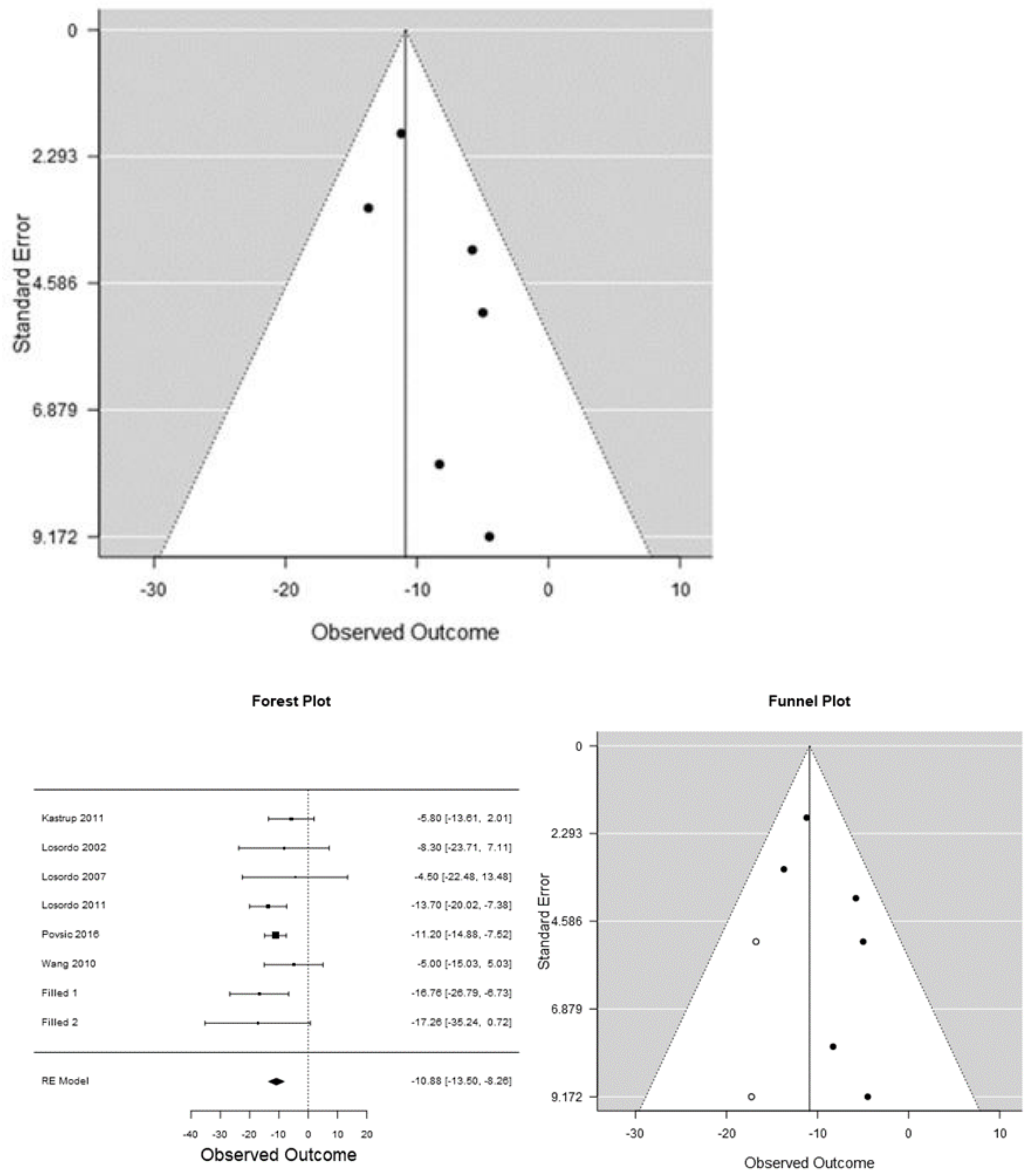

Supplementary Figure 10: Funnel plot for the analysis of absolute mean difference of angina episodes (upper panel); Trim-fill analyses with the addition of 2 estimates and the resulting forest plot (lower panel).

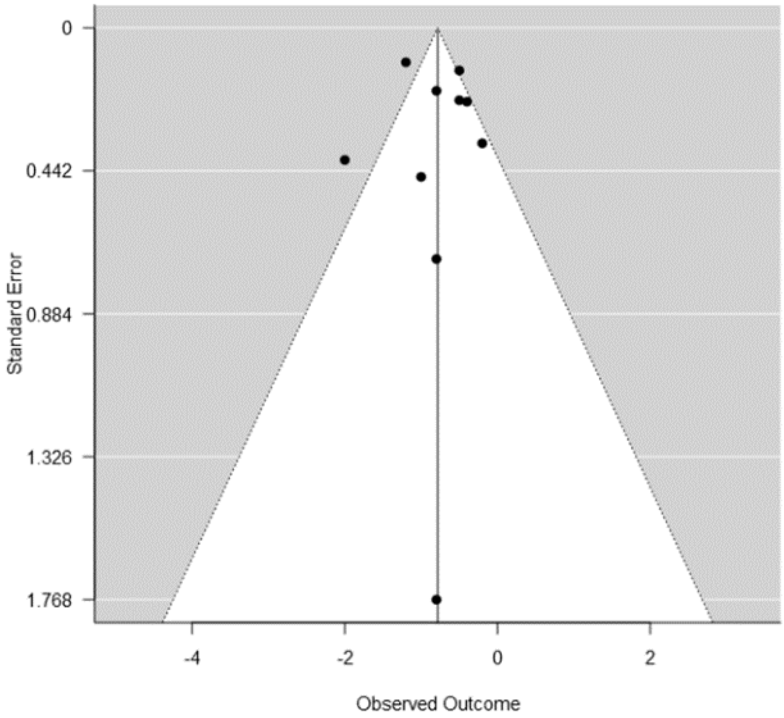

Supplementary Figure 11: Funnel plot for the analysis of CCS class; The trim-fill analysis did not add any further study.

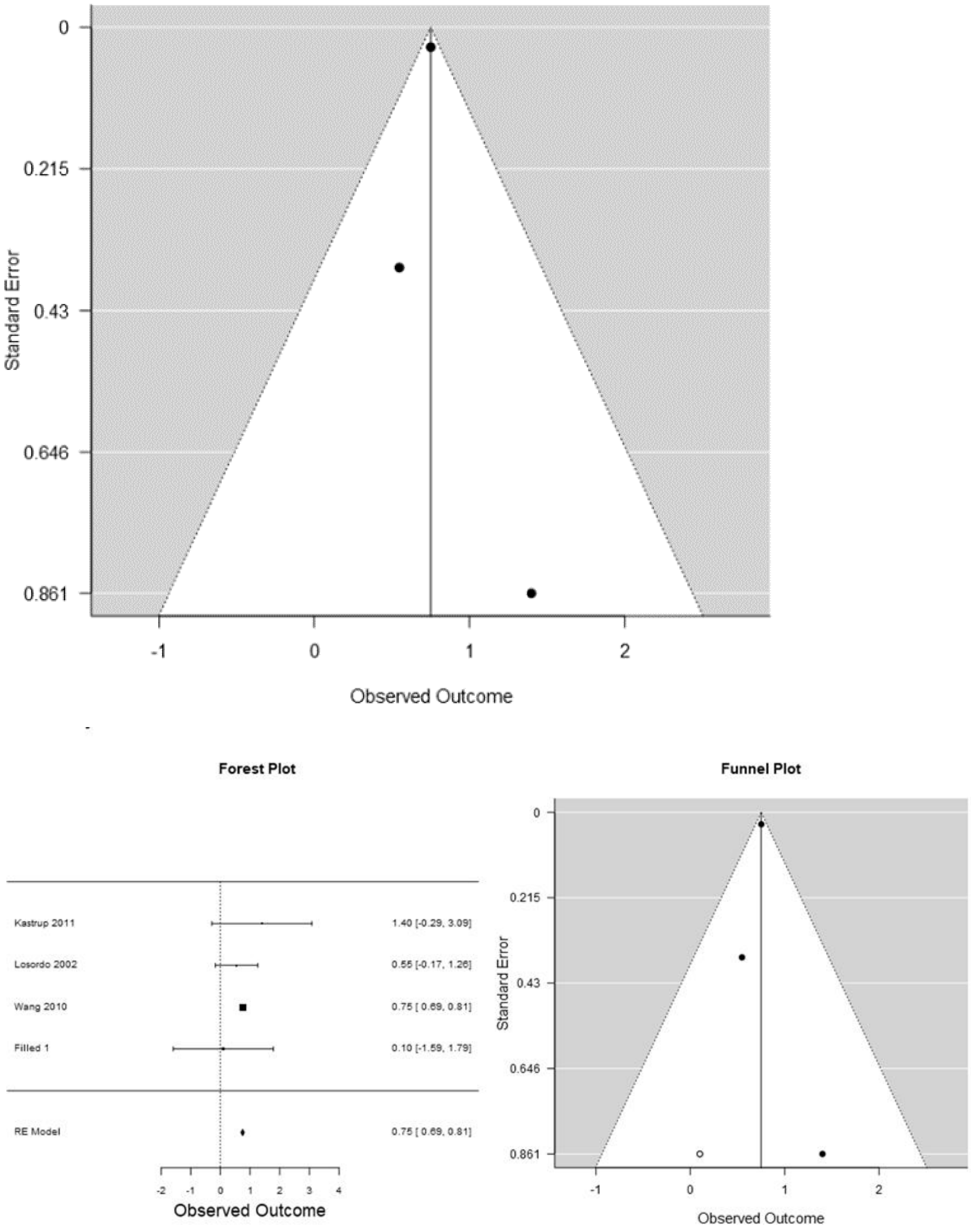

Supplementary Figure: Funnel plot for the analysis of relative change in NTG use (upper panel); Trim-fill analyses with the addition of 1 estimate and the resulting forest plot (lower panel).

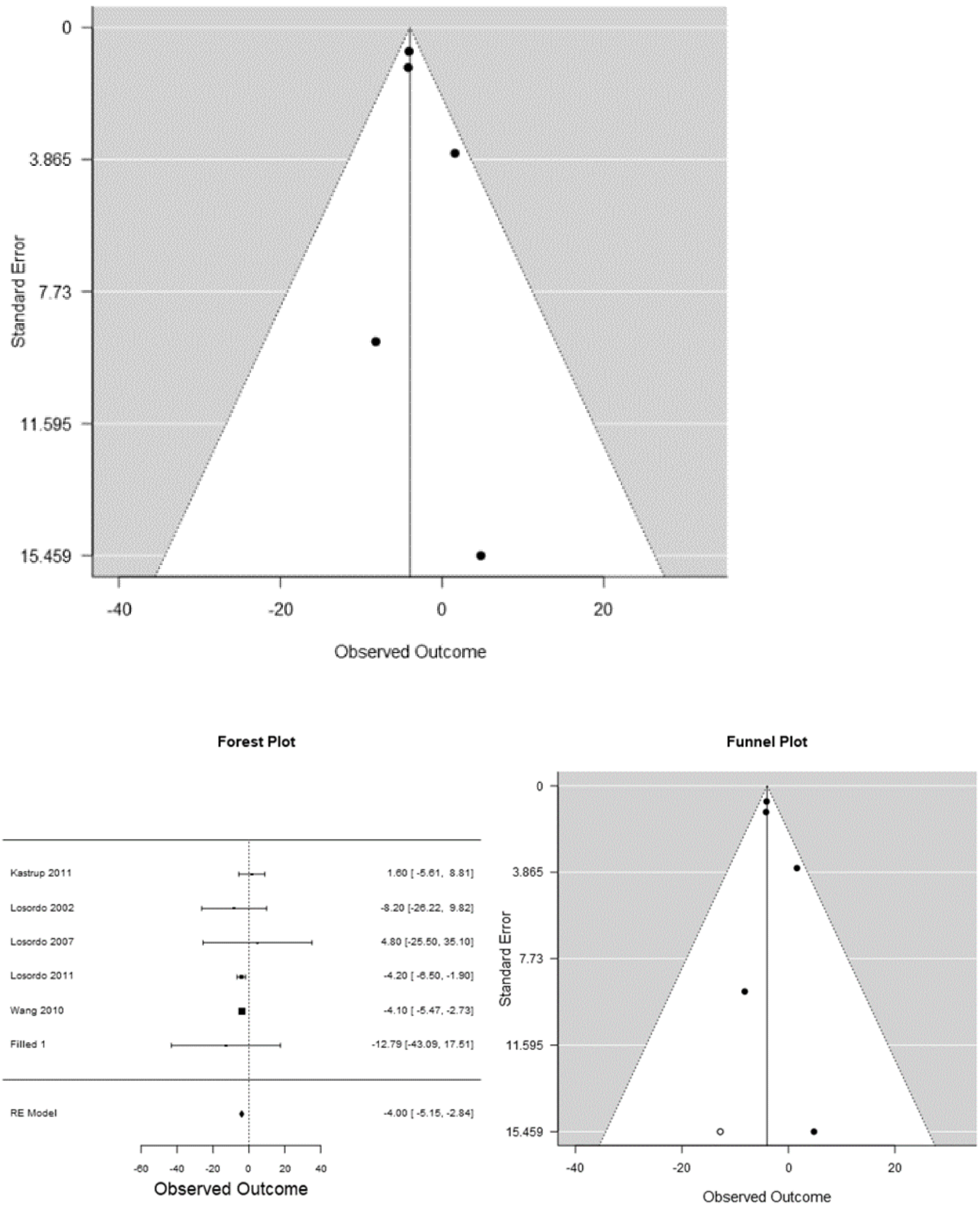

Supplementary Figure: Funnel plot for the analysis of mean difference of NTG use (upper panel); Trim-fill analyses with the addition of 1 estimate and the resulting forest plot (lower panel).

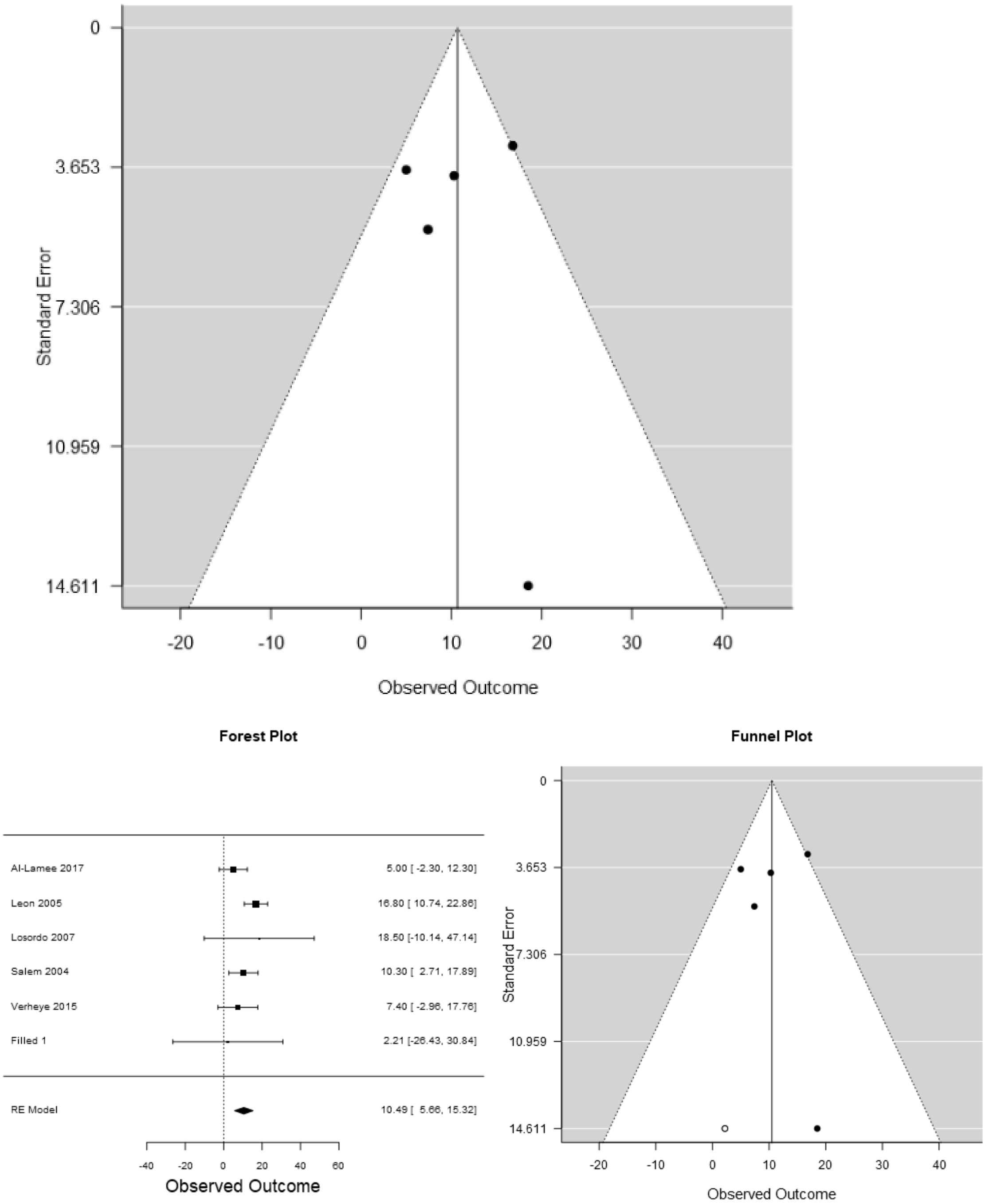

Supplementary Figure: Funnel plot for the analysis of SAQ physical functioning (upper panel); Trim-fill analyses with the addition of 1 estimate and the resulting forest plot (lower panel).

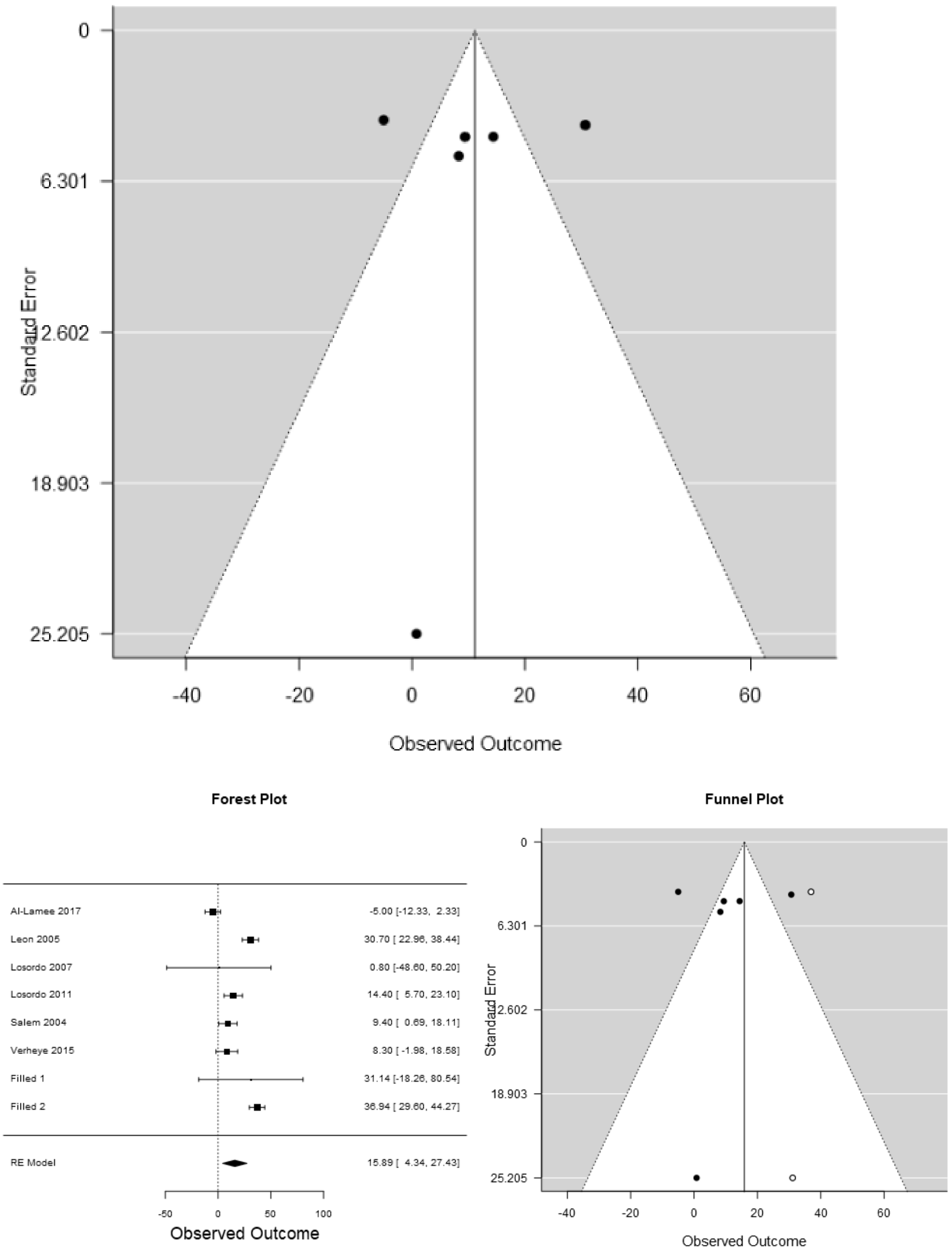

Supplementary Figure: Funnel plot for the analysis of SAQ anginal stability (upper panel); Trim-fill analyses with the addition of 1 estimate and the resulting forest plot (lower panel).

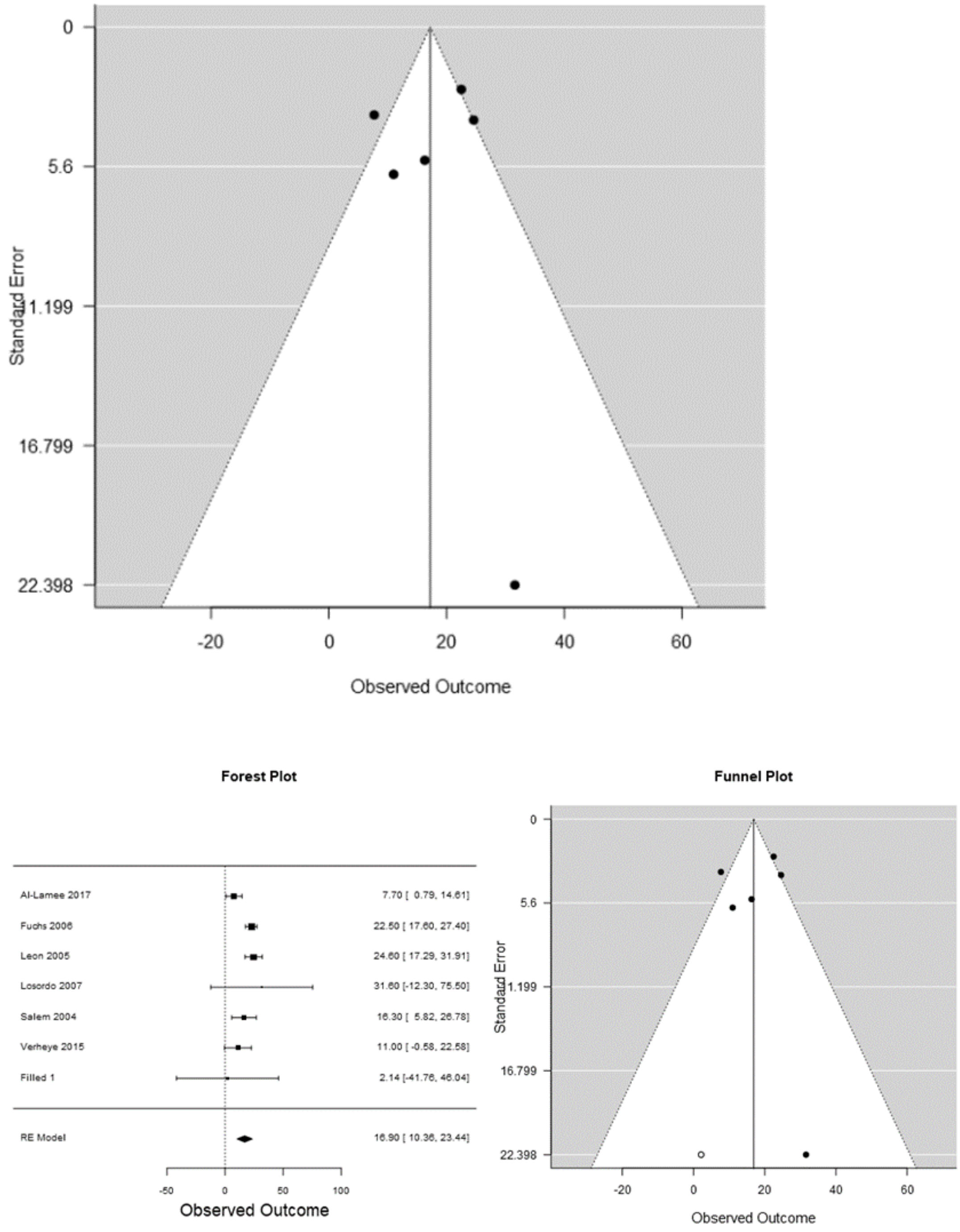

Supplementary Figure: Funnel plot for the analysis of SAQ anginal frequency (upper panel); Trim-fill analyses with the addition of 1 estimate and the resulting forest plot (lower panel).

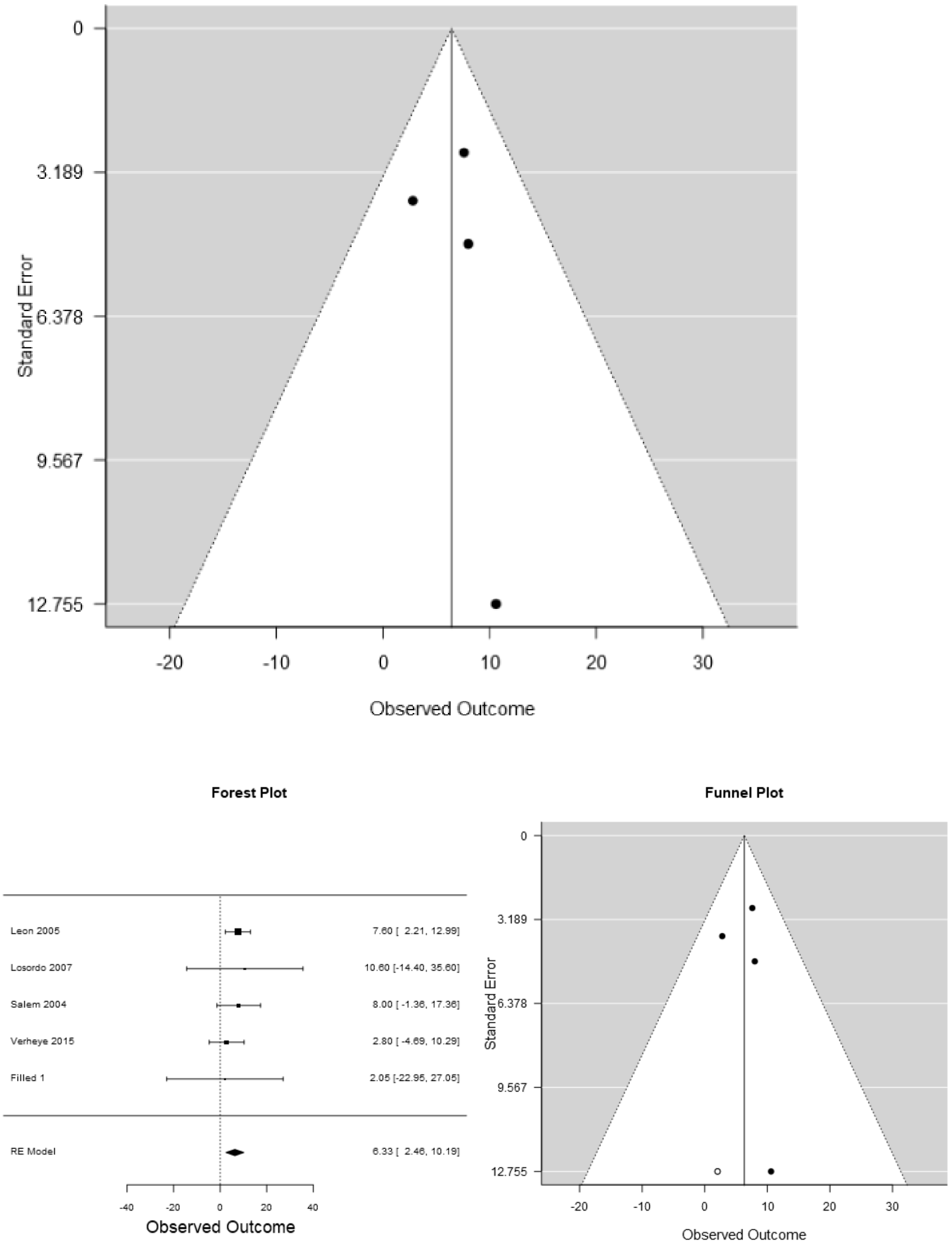

Supplementary Figure: Funnel plot for the analysis of SAQ treatment satisfaction (upper panel); Trim-fill analyses with the addition of 1 estimate and the resulting forest plot (lower panel).

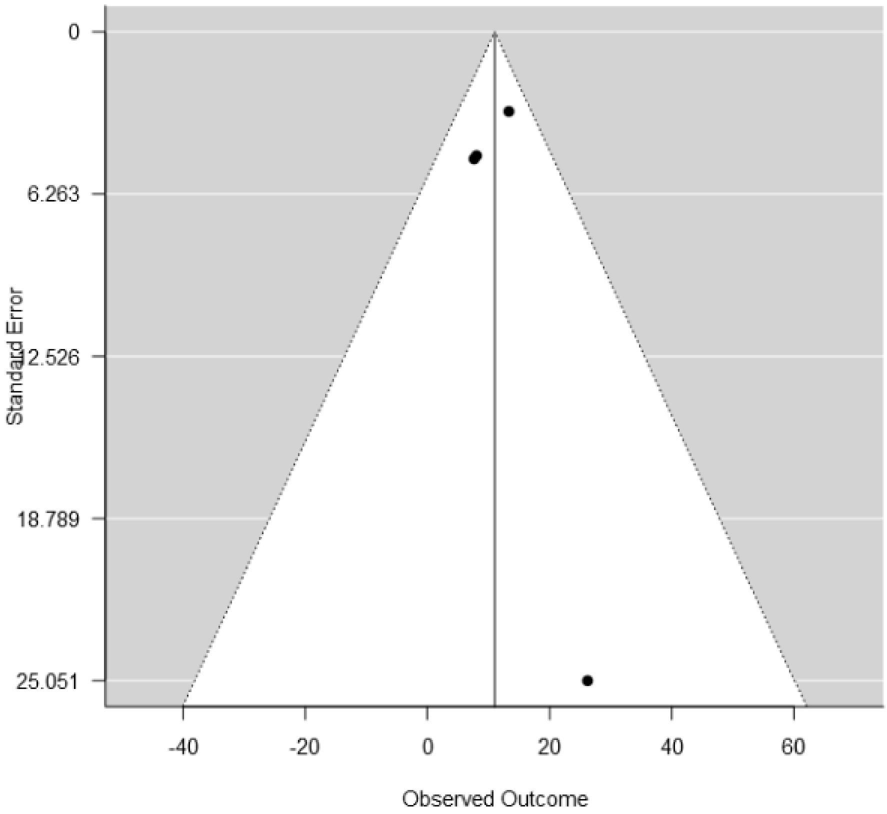

Supplementary Figure: Funnel plot for the analysis of SAQ disease perception (upper panel); The trim-fill analysis did not add any further study.
