# Supplementary material for: The sham effect of invasive interventions in chronic coronary syndromes: a systematic review and meta-analysis
Source: BMC Cardiovasc Disord. 2022 May 14;22:223. doi: 10.1186/s12872-022-02658-x (PMC9107755; doi:10.1186/s12872-022-02658-x)
Supplement: Supplementary file 4 — Additional file 4: Sensitivity analyses and meta-regressions. [file 12872_2022_2658_MOESM4_ESM.pdf]

ADDITIONAL FILE 4

SENSITIVITY ANALYSES AND META-REGRESSIONS

The sham effect of invasive interventions in chronic coronary syndromes – a systematic review and meta-analysis

Authors: Catarina Palma, Cláudio David MD, Ricardo M Fernandes MD PhD, Fausto J Pinto MD PhD, João Costa MD PhD, Joaquim J Ferreira MD PhD, Daniel Caldeira MD PhD

Table of contents

Supplementary Figure: Analysis of CCS class mean change according with the type of sham 3

Supplementary Figure: Analysis of relative change in angina episodes according with the mean LVEF/proportion of HF patients. HF: Heart failure; NR: not reported; NOTE: all these trials had sham intervention+placebo-like solution 3

Supplementary Figure: Analysis of CCS class mean change according with the mean LVEF/proportion of HF patients. HF: Heart failure; NR: not reported 4

Supplementary Figure: Analysis of absolute NTG use mean change according with the mean LVEF/proportion of HF patients. HF: Heart failure; NR: not reported 4

Supplementary Figure: Analysis SAQ angina stability mean change according with the type of sham 5

Supplementary Figure: Analysis of SAQ angina stability mean change according with the mean LVEF/proportion of HF patients. HF: Heart failure; NR: not reported 5

Supplementary Figure: Analysis SAQ angina frequency mean change according with the type of sham 6

Supplementary Figure: Analysis of SAQ angina frequency mean change according with the mean LVEF/proportion of HF patients. HF: Heart failure; NR: not reported 6

Supplementary Figure: Analyses of CCS class and SAQ angina stability/frequency mean change excluding studies with some concerns in risk of bias. 7

Supplementary Figure: Analyses of relative change in angina episodes excluding studies with some concerns in risk of bias. 7

Supplementary Figure: Meta-regression for mean age and relative change in exercise time in the sham arm. 8

Supplementary Figure: Meta-regression for percentage of diabetes and relative change in exercise time in the sham arm. 8

Supplementary Figure: Meta-regression for percentage of male patients and relative change in exercise time in the sham arm. 9

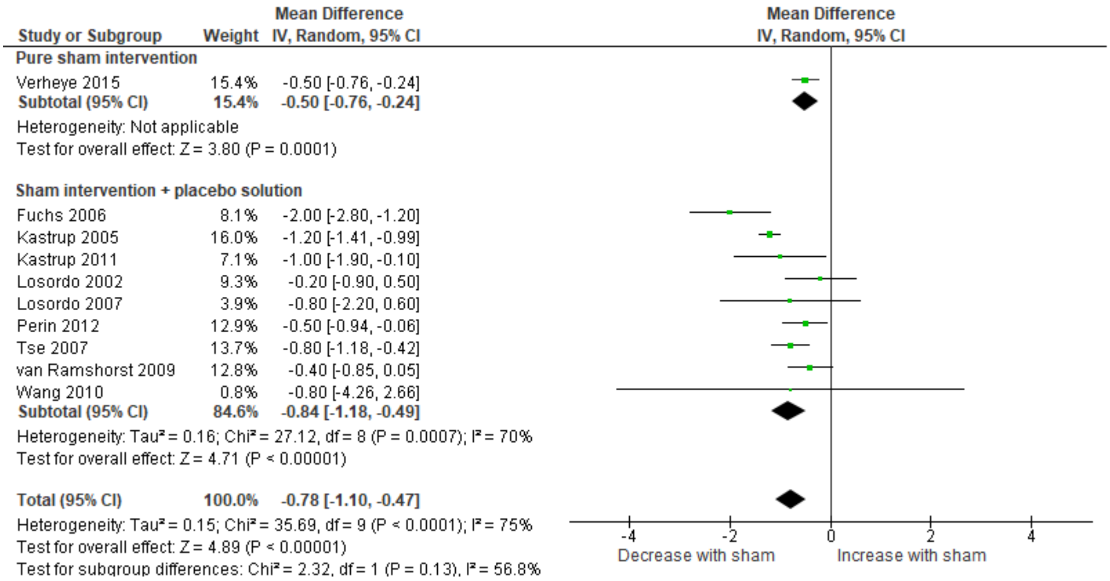

Supplementary Figure: Analysis of CCS class mean change according with the type of sham

Pooled and individual relative effect and 95% CI for exercise time according with the type of sham (pure sham intervention or sham intervention + placebo solution).

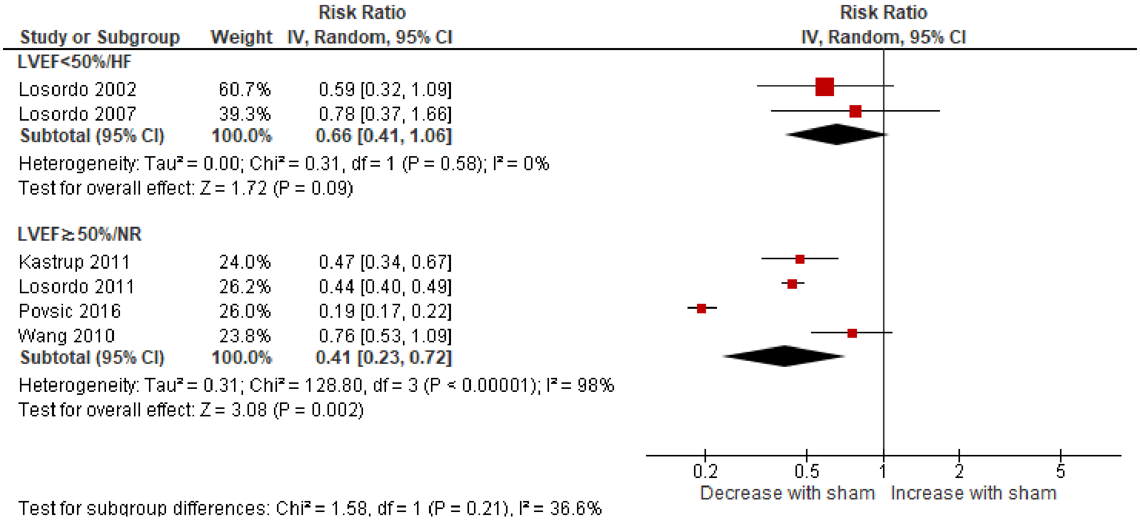

Supplementary Figure: Analysis of relative change in angina episodes according with the mean LVEF/proportion of HF patients.

HF: Heart failure; NR: not reported; NOTE: all these trials had sham intervention+placebo-like solution

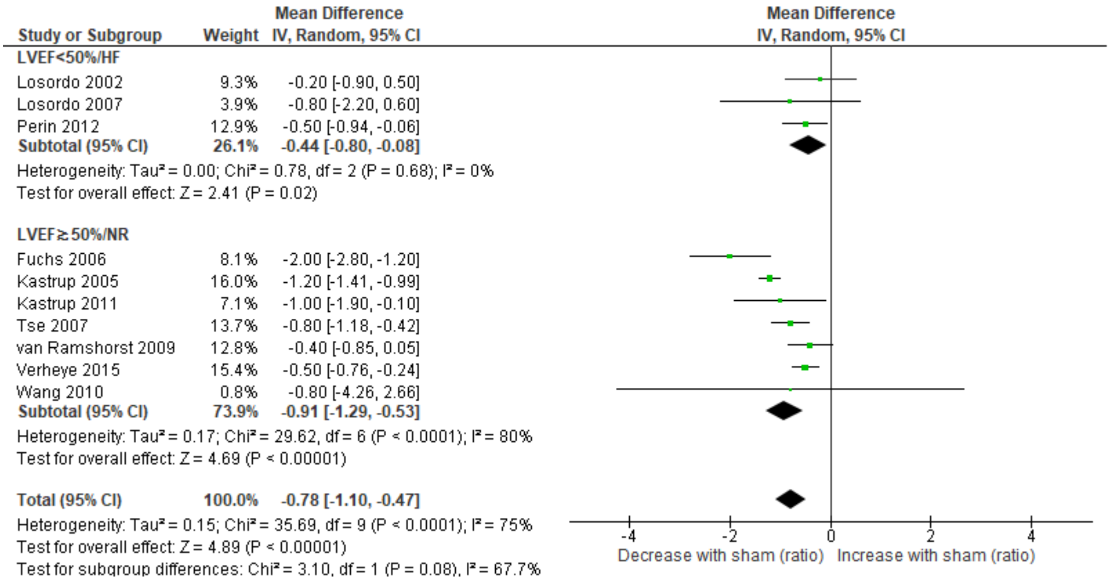

Supplementary Figure: Analysis of CCS class mean change according with the mean LVEF/proportion of HF patients. HF: Heart failure; NR: not reported

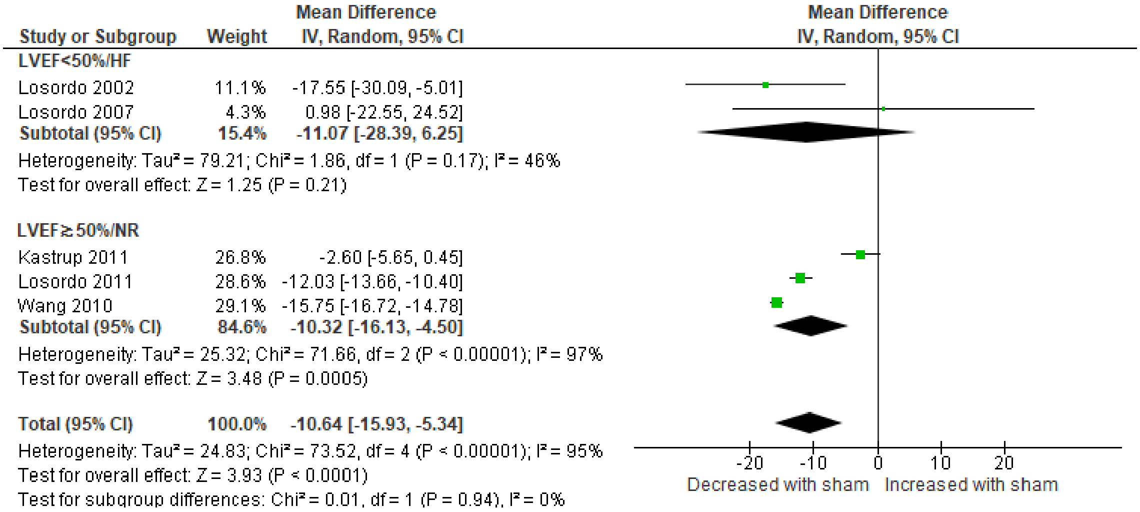

Supplementary Figure: Analysis of absolute NTG use mean change according with the mean LVEF/proportion of HF patients. HF: Heart failure; NR: not reported

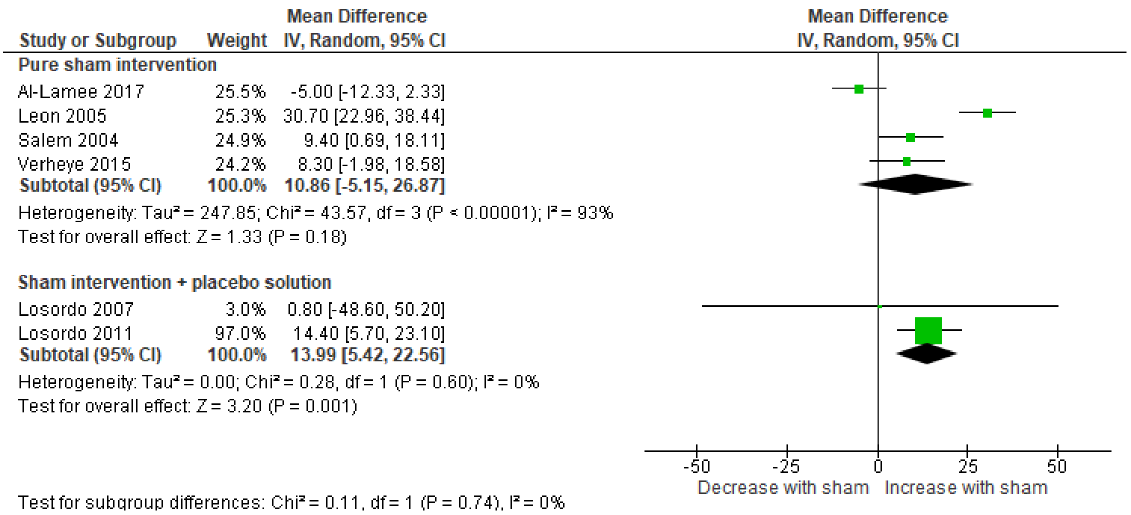

Supplementary Figure: Analysis SAQ angina stability mean change according with the type of sham

Pooled and individual relative effect and 95% CI for exercise time according with the type of sham (pure sham intervention or sham intervention + placebo solution).

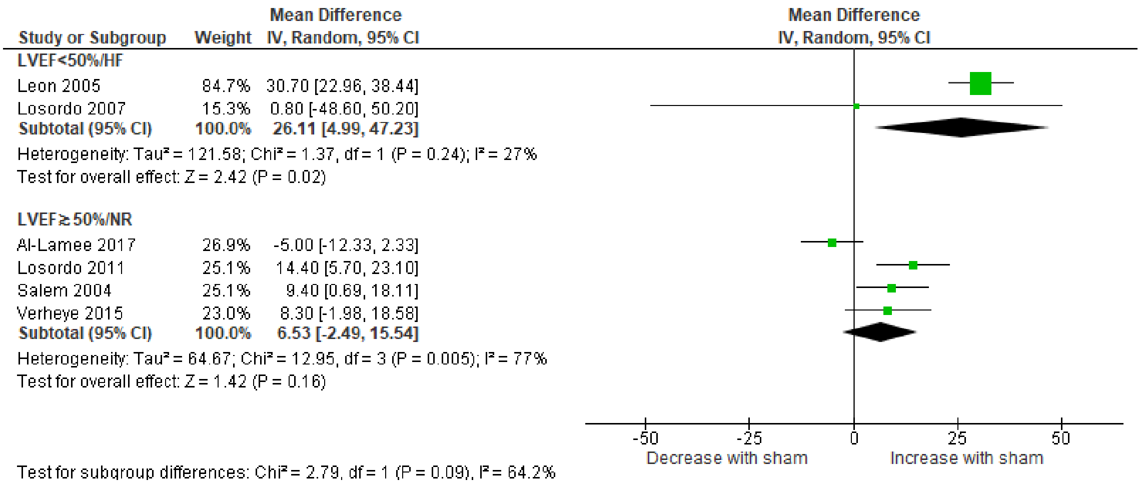

Supplementary Figure: Analysis of SAQ angina stability mean change according with the mean LVEF/proportion of HF patients. HF: Heart failure; NR: not reported

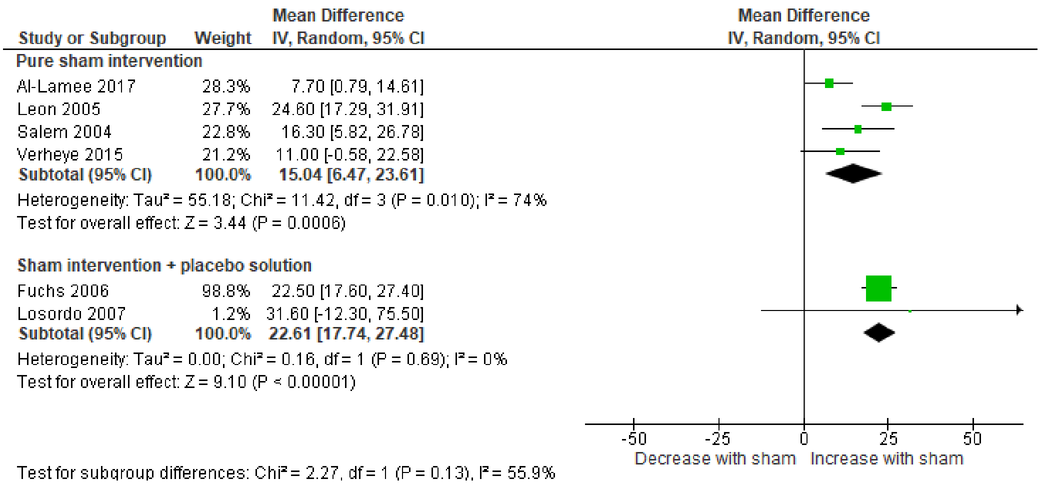

Supplementary Figure: Analysis SAQ angina frequency mean change according with the type of sham

Pooled and individual relative effect and 95% CI for exercise time according with the type of sham (pure sham intervention or sham intervention + placebo solution).

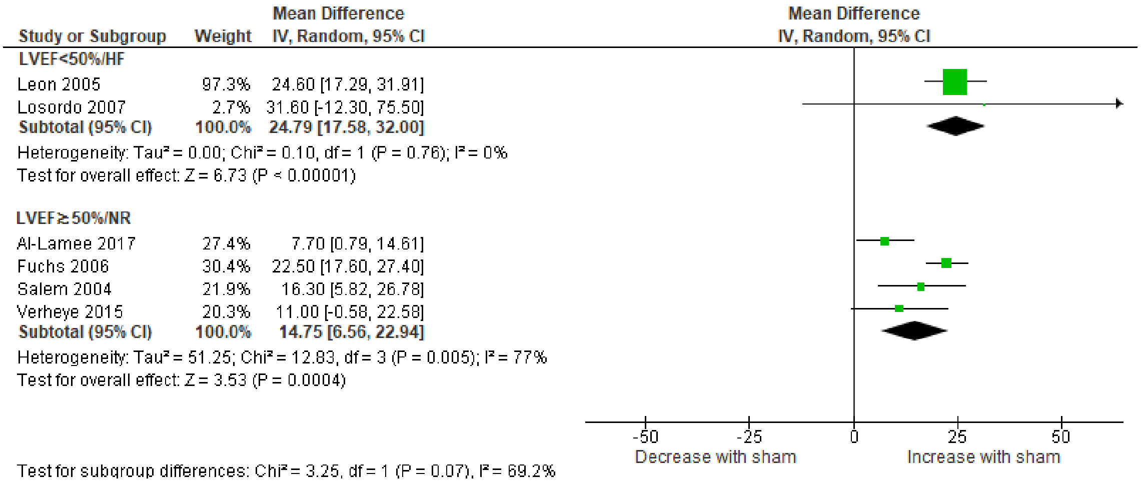

Supplementary Figure: Analysis of SAQ angina frequency mean change according with the mean LVEF/proportion of HF patients.

HF: Heart failure; NR: not reported

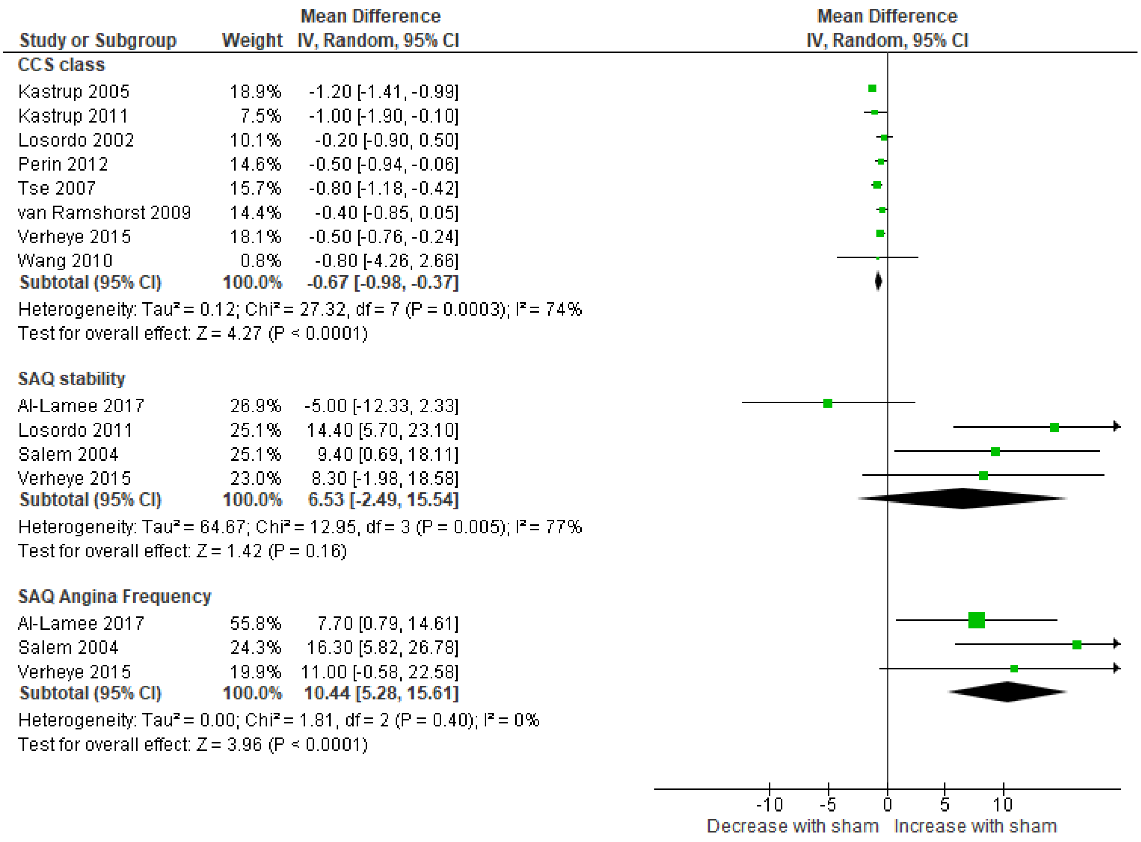

Supplementary Figure: Analyses of CCS class and SAQ angina stability/frequency mean change excluding studies with some concerns in risk of bias.

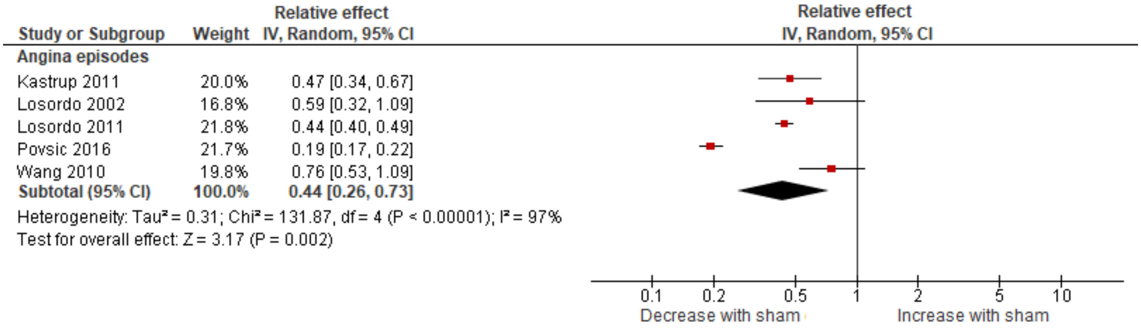

Supplementary Figure: Analyses of relative change in angina episodes excluding studies with some concerns in risk of bias.

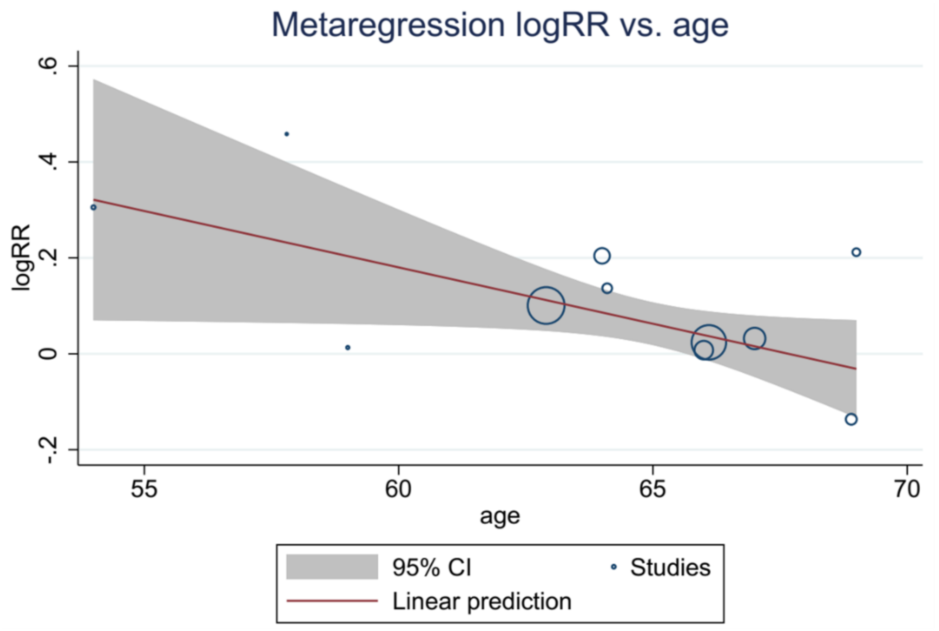

variable	Coefficient	Std. err.	z	P>|z|	[95% conf. interval]	
age	-.0235061	.0115204	-2.04	0.041	-.0460857	-.0009266

Supplementary Figure: Meta-regression for mean age and relative change in exercise time in the sham arm.

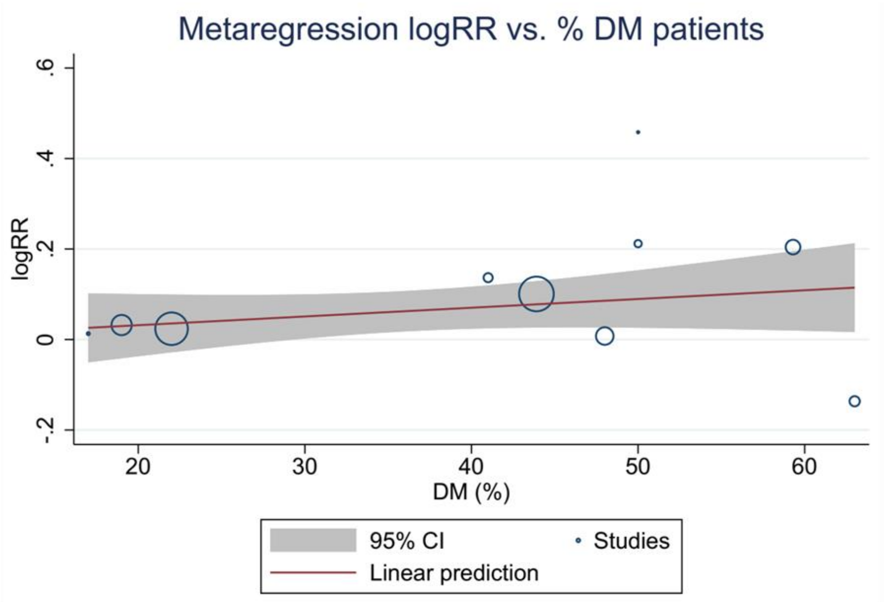

variable	Coefficient	Std. err.	z	P>|z|	[95% conf. interval]	
DM	.0019309	.0016551	1.17	0.243	-.001313	.0051748

Supplementary Figure: Meta-regression for percentage of diabetes and relative change in exercise time in the sham arm.

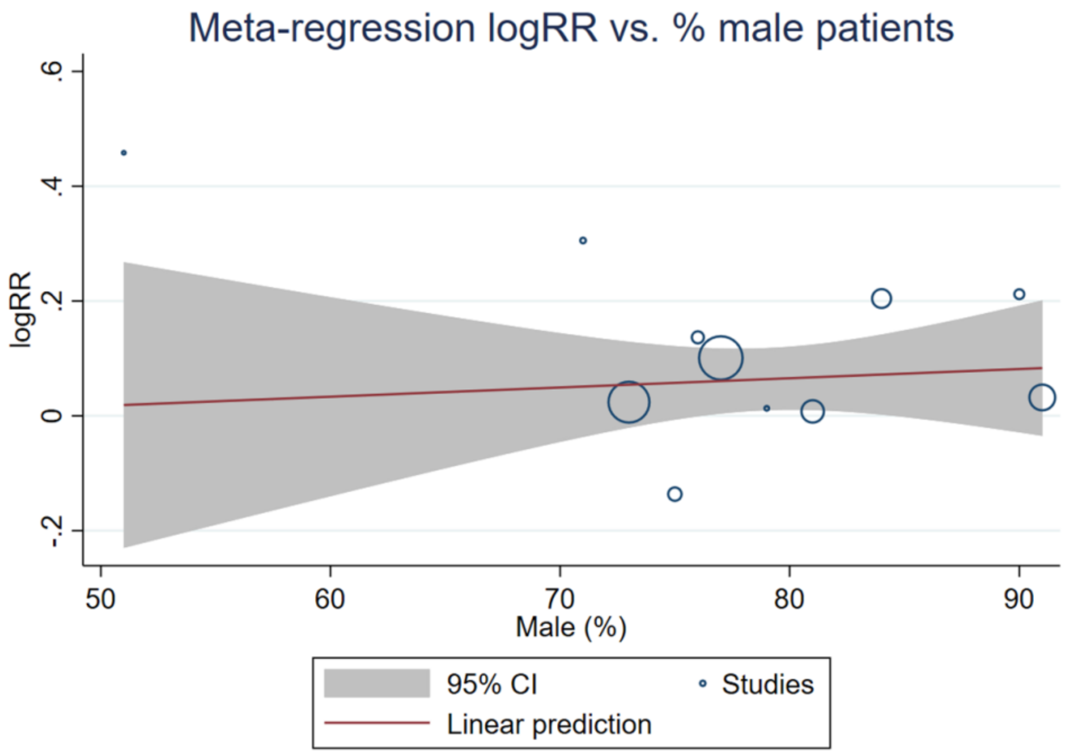

Variable	Coefficient	Std. err.	z	P>|z|	[95% conf. interval]	
Male	.001608	.0044341	0.36	0.717	-.0070826	.0102986

Supplementary Figure: Meta-regression for percentage of male patients and relative change in exercise time in the sham arm.
